# Development of a survey tool to assess the environmental determinants of health-enabling food retail practice in Aboriginal and Torres Strait Islander communities of remote Australia

**DOI:** 10.1186/s12889-024-17945-9

**Published:** 2024-02-12

**Authors:** Emma van Burgel, Molly Fairweather, Amanda Hill, Meaghan Christian, Megan Ferguson, Amanda Lee, Sarah Funston, Bronwyn Fredericks, Emma McMahon, Christina Pollard, Julie Brimblecombe

**Affiliations:** 1 Department of Nutrition, Dietetics and Food, Monash University, Melbourne, VIC 3168 Australia; 2 Menzies School of Health Research, Royal Darwin Hospital Campus, Darwin, NT Australia; 3 School of Public Health, The University of Queensland, Brisbane, QLD Australia; 4 Arnhem Land Progress Aboriginal Corporation, Darwin, NT Australia; 5 School of Population Health, Curtin University, Perth, WA Australia

**Keywords:** Aboriginal, Indigenous, Environment and public health, Food security, Food retail, Remote store

## Abstract

**Background:**

Environmental factors can impact the ability of food retail businesses to implement best practice health-enabling food retail.

**Methods:**

We co-designed a short-item survey on factors influencing food retail health-enabling practice in a remote Australian setting. Publicly available submissions to an Australian Parliamentary Inquiry into food pricing and food security in remote Indigenous communities were coded using an existing remote community food systems assessment tool and thematically analysed. Themes informed survey questions that were then prioritised, refined and pre-tested with expert stakeholder input.

**Results:**

One-hundred and eleven submissions were coded, and 100 themes identified. Supply chain related data produced the most themes (*n* = 25). The resulting 26-item survey comprised questions to assess the perceived impact of environmental factors on a store’s health-enabling practice (*n* = 20) and frequency of occurrence (*n* = 6).

**Conclusions:**

The application of this evidence-informed, co-designed survey will provide a first-time cross-sectional analysis and the potential for ongoing longitudinal data and advocacy on how environmental factors affect the operations of remote stores.

**Supplementary Information:**

The online version contains supplementary material available at 10.1186/s12889-024-17945-9.

## Introduction

Retail food environments can foster healthy food and drink purchases through the use of retail merchandising and marketing practices and thereby support healthy diets [1]. However, retail food environments more commonly promote unhealthy food and drink options and less nutritious dietary intakes. Unhealthy diets are a lead contributor to the global burden of noncommunicable disease, including type 2 diabetes, cancer and cardiovascular disease [2]. This burden of disease inequitably impacts remote dwelling populations and Indigenous Peoples. In Australia, Aboriginal and Torres Strait Islander Peoples in remote and very remote communities experience a higher burden of disease (486 Disability-adjusted life years (DALY) per 1000 population and 492 DALY per 1000 population, respectively) than that of Aboriginal and Torres Strait Islander Peoples in major cities (393 DALY per 1000 population) [3]. Aboriginal and Torres Strait Islander Peoples in all of Australia experience a burden of disease 2.3 times higher than that of non-Indigenous Australians [4]. An estimated 34% of this disparity is due to socio-economic differences [4]. The primary supplier of purchased food and drink in a remote Aboriginal and Torres Strait Islander community is the local food retail store [5]. Therefore, it is imperative that remote retail food environments are shaped to be conducive to health.

Product price, availability, placement and promotion, also known as the ‘Marketing Mix’ or the ‘4Ps’, influences food options and customer purchasing behaviour [6]. The 4Ps have successfully been used to support health-enabling practice in retail food environments by modifying merchandising practice to promote the sale of healthy food and drinks, and limit the promotion of unhealthy food and drinks [7–12]. Such practice influences consumer purchasing through strategies such as price promotions on fruits and vegetables [8], modifying the layout of a store to have healthy food and drink products at the front of the store [9], stocking requirements for specific healthy food and drinks [10], restricting the promotion of unhealthy food and drinks [11], and promoting healthy foods with the use of shelf-tags [12].

A wide range of factors can influence the implementation of health-enabling food retail practices. An understanding of these is important to help shape strategies to support implementation success and thereby intervention impact for improved population nutrition. Several systematic reviews have summarised the evidence related to these factors across a range of settings, however, the majority of studies reviewed have focused on studies in the United States of America (USA) and on the in-store environment [13–15]. Retail food environments across the world are diverse in type, ranging from street stalls to hypermarkets, and in the location of populations served, from those in cities to those in very remote locations. The retail food landscape of remote Australia comprises of over 200 retail food stores in communities that are classified as remote or very remote in terms of access to services [16] and that serve a largely Aboriginal and Torres Strait Islander population of approximately 123,300 people [17, 18]. These stores operate alongside a traditional Indigenous food system including, but not limited to, hunting and gathering on traditional lands, and the care and management of traditional lands and waterways [19]. The different ownership and management structures of these stores, including Aboriginal corporation owned or privately owned and store group managed or independently managed, can influence store operations, including buying and market power [20]. Despite strong community ownership driving store policy to support community food security, the remote food supply is vulnerable to shocks. Geographical distance from supply centres and weather events can influence store operations [21], with many store businesses in remote Australia operating in isolated environments. Goods must be freighted from suppliers over long distances by road, rail, sea or air, and adverse weather events such as flooding, cyclones, and seasonal weather conditions, including extreme heat and humidity, can make it difficult to stock, replenish and store perishable goods [22]. These factors can contribute to limited stock availability, higher risk of lower quality goods, and prices being consistently higher than those in metropolitan areas [23, 24]. A government food price survey in 2021 found a healthy food basket in communities in remote Northern Territory (NT) of Australia to cost 52% more in remote stores when compared to regional centre supermarkets [25, 26].

There is some evidence in Australia on the determinants of individual-level food behaviour in the remote community context that includes the influence of the store environment [27–29]. However, there is a dearth of evidence on the multi-level socio-ecological determinants of community store retail practices, including the physical and natural environments, that in turn then shapes individual-level food behaviour [30]. For example, the vulnerability of remote food retail and its food supply is known anecdotally to have a strong influence on store health-enabling practice, however there is little empirical evidence of this nor has there been an investigation of these influences across a wide number of communities. The following study aimed to develop a short-item survey to collect information from store owners and/or managers on the perceived level of influence of broader socio-ecological (environmental) factors on their ability to implement best practice policy that enables healthy food purchasing in their stores. Remote food retail is part of the wider food system, and this study could assist in understanding why certain initiatives are effective, or not, and could provide important opportunities to optimise the effectiveness of interventions to improve the remote food retail environment, promote stability in the food security of remote communities, and support healthier diets for populations living remotely.

## Methods

This study was part of a wider project, Benchmarking for Healthy Stores in Aboriginal and Torres Strait Islander remote communities, which is investigating the impact of a continuous quality improvement approach that benchmarks health-enabling policy and practice, and ultimately purchasing, in remote stores. The resulting short-item survey (survey) is to be administered once a year during the 3-year project with 29 remote stores in the NT, Australia, with the intention to extend its use by public health practitioners with all remote Australian community stores in the future. In this study, we refer to store owners and/or managers as the decision makers for each store, and we recognise that food retail behaviour and therefore practice is influenced by not only individual-level factors (e.g., customers and retailers) and the setting (store organisation) but by many socio-ecological factors operating at multiple levels of influence including in the domain of the individual, the organisation, the community and the wider natural, built, cultural and social environments [14, 30–32]. By surveying the decision-makers of the store, this study aims to give insight into the socio-ecological barriers and enablers for implementation of health-enabling practices. It will help to generate valuable evidence on the influence of socio-ecological (environmental) determinants on health-enabling store practices.

**Fig. 1:**
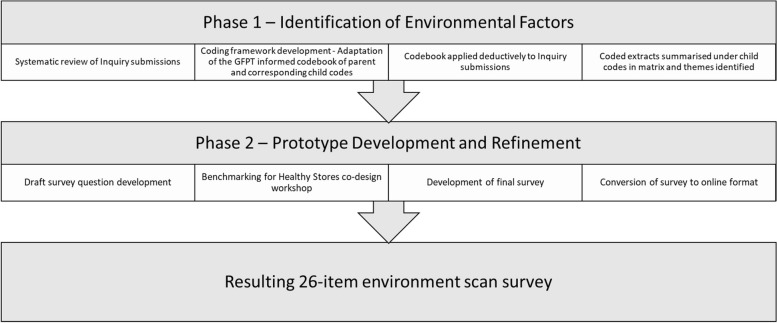
Flowchart of study methods. GFPT: Good Food Planning Tool

A two-phased multi-method co-design approach was used that involved: 1) identification of environmental factors through systematic document review and expert consultation; and 2) survey prototype development and refinement through expert review, a face-to-face stakeholder workshop, and pre-testing (see Fig. 1). An environment scan task group (task group) was established to provide expert input into survey development. This group comprised of 7 members including academics with extensive experience in remote store public health research, a store group public health nutritionist and a food supply nutritionist working for an Aboriginal Health Service in remote NT, Australia.

### Phase 1: Identification of environmental factors

This phase followed the Gale et al. Framework Method of management and analysis of qualitative data, producing a matrix output from the submissions, codes and themes to guide the survey prototype development [33]. Stage 1 (transcription) of The Framework Method was not applicable to this study as it analysed existing documents [33]. Stage 2 (familiarisation) involved systematic review of submissions to the 2020 Australian Government Parliamentary Inquiry into food pricing and food security in remote Indigenous communities (the Inquiry). Stages 3 and 4 (developing an analytical framework and applying the framework) involved consultation with the task group through regular meetings for provision of expert advice, and development of a codebook informed by an existing remote community food systems planning tool, the Good Food Planning Tool (GFPT), describing socio-ecological factors affecting the food system in remote communities [19, 31, 33]. Stage 5 (charting the data) involved subsequent application of the codebook to the submissions and the creation of a matrix output.

#### Familiarisation

Publicly available submissions on the Inquiry website from organisations or individuals concerned with food retail in remote Aboriginal and Torres Strait Islander communities were read by two researchers (EvB and MFa) to ensure familiarisation [22].

#### Codebook development

The GFPT was developed by researchers from Menzies School of Health Research with four remote Aboriginal communities and a total of 148 stakeholders (including 78 Indigenous residents from remote communities) through literature review, a series of workshops and field testing [19]. This participatory process included systematic identification of the range of activities in the Indigenous Australian remote community context that related to each of the dimensions of food security (food availability, access and utilisation) for each function of a food system (food collection/growing to waste disposal and recycling) and in each area of the socio-ecological environment (socio-cultural, physical, economic and political and natural) [19]. It therefore captures the array of practices to address the determinants for a food secure community food system in remote Aboriginal and Torres Strait Islander communities across five system domains: Food Businesses, Strong Leadership and Partnerships, Community and Services, Traditional Foods and Local Food Production, and Buildings, Public Places and Transport [19]. Under each of the system domains, the GFPT includes a number of activity areas and associated discussion points (best practice characteristics) [19]. To determine the GFPT areas directly applicable to the food retail practice, each of the ‘discussion points’ within the GFPT (*n* = 120) were assessed against a set of criteria constructed by the task group to determine if the ‘discussion point’ described factors which could have a direct impact, or an indirect but potentially substantial impact, on a store’s health-enabling practice (Table 1). For instance, under the GFPT ‘Strong Leaderships and Partnerships’ domain, the discussion point “People who provide a service for the community are valued” was included with the rationale that if those involved with remote store practice are valued by the community, they are more likely to be motivated and willing to implement health-enabling practice and therefore could have a direct impact. Under the ‘Community and Services’ domain, the discussion point “Nutrition and health learning is integrated into school curricula” was identified as having an indirect but potentially substantial impact on health-enabling practice through community demand and capacity building. Those ‘discussion points’ that were deemed as having an indirect and potentially insubstantial impact on a store’s health-enabling practice were removed from the 120 ‘discussion points’ used for the study. For example, under the GFPT ‘Buildings, Public Places and Transport’ domain, the discussion point “suitable public infrastructure to support healthy eating and wellbeing” was excluded as the store does not directly or substantially interact with this context.

**Table 1:** Inclusion/exclusion criteria to identify factors considered to influence health-enabling store practice

Area of impact	Include	Exclude
Context	-Remote Aboriginal and Torres Strait Islander communities -Those involved in service delivery for/to remote communities -Remote food retail business (i.e., store, takeaway, roadhouse)	-General Australian population -Land management, waste disposal and pest control (note: pest control in store context is included) -Food relief services -Provision of foods in other settings (i.e. aged care, food relief) -Provision of foods in stores other than in remote communities -Public infrastructure
Price	Natural, physical, human and/or social/cultural factors that directly influence product pricing policy OR could have a substantial impact on pricing via an indirect mechanism - Funding/subsidies/grants -Competition/competitors -Community demand -Weather events/natural disasters/pandemics -Freight costs/time/frequency -Contracts with suppliers - Price from supplier/supply options	
Promotion	Natural, physical, human and/or social/cultural factors that directly influence product promotion OR could have a substantial impact on promotion via an indirect mechanism -Established healthy eating campaigns/consistency of healthy eating campaigns -Partnerships with the remote store including nutrition workforce, healthcare and schools -Government (local, state, federal) policies influencing store practice	-Other policies not directly related to food/nutrition promotion
Placement	Natural, physical, human and/or social/cultural factors that directly influence how/where products are placed in-store and policy relating to this OR could have a substantial impact on placement via an indirect mechanism -Store size -Store layout / architecture -Contracts with suppliers	
Product	Natural, physical, human and/or social/cultural factors that directly influence which products are made available in-store and policy relating to this OR could have a substantial impact on product availability via an indirect mechanism -Community involvement with the store and relationships with store -Product availability -Workforce – skills and abilities, values, support, training, management, knowledge -Price of goods -Store facilities and equipment and storage -Delivery of goods (time, cost, frequency, mode, quality of goods on arrival)	-Locally produced food not sold in store -Traditional food procurement -Resources and equipment for local food procurement/other goods other than food sold in remote stores

Included discussion points were then grouped into sub-headings related to components of store practice or operations (e.g., supply chain, workforce and staffing, governance and management), after discussion and consensus with the task group. These sub-headings of socio-ecological determinants of store health-enabling practice then informed the codebook of parent codes and their corresponding child codes, developed by MFa (see Additional file 1: Supplementary Table 1) with input from JB and review by the task group. The task group provided expert guidance and context to the codebook through discussion and consensus.

#### Applying the framework

The Inquiry submissions were uploaded into Computer Assisted Qualitative Data Analysis Software (CAQDAS; NVivo, Release 1.6, QSR International, Melbourne, Australia). Two researchers (EvB, MFa) coded three submissions independently to ensure consistent application of the codebook. Coding was compared at this stage using an ‘eyeball’ approach, where discrepancies were discussed, and agreement reached. Any unresolved discrepancies were reviewed and resolved by the senior author (JB). Resulting changes to the codebook from the trial coding were completed before all remaining submissions were coded deductively by one researcher (EvB or MFa), resolving any queries through discussion and consensus between both researchers.

#### Charting the data

Coded submission data were indexed under their child codes (e.g., the child code of ‘Management values and leadership’ under the parent code of Store management, governance and decision-making) from the codebook using the Reports function in the CAQDAS. Using this dataset, themes and subthemes were generated inductively by two authors (EvB, MFa), under each of the child codes, by identifying patterns in the coded data (see Additional file 1: Supplementary Table 2). For example, extracted text “store management groups valuing social returns over commercial gains” was themed as “Food security and wellbeing of the community”, with a sub-theme of “store management groups’ profits being funnelled back into the community to improve wellbeing”. Data within each child code were then summarised into a matrix developed in Microsoft Excel (version 16.43, Washington), consisting of rows of summarised data extracted from the submissions and columns of themes and subthemes. The matrix also included each theme’s frequency of occurrence in the data. Following discussion with the task group, the rows of summarised text data were colour-coded to visually identify patterns in the data and thus define the overarching themes and subthemes for each of the original child codes.

### Phase 2: Prototype development and refinement

This phase involved the development of draft survey questions in conjunction with the expertise of the task group. It also involved a face-to-face workshop for the purpose of co-designing and refining the survey for use with remote community store representatives. A survey was chosen as the data collection tool to minimise the time cost to store owners/managers, which has been previously identified as a structural barrier to healthy food-store interventions [14].

#### Question development

Draft survey questions (1–3 per child code) were developed by two researchers (MFa, EvB), with input from the senior author (JB). Each child code matrix revealed themes and subthemes (corresponding to a child code) as described above, with the most frequently mentioned themes derived from the submission data being converted into a question. Questions were presented as a statement pertaining to the identified environmental factor, and asked respondents to select “to what extent do you agree?” on a 5-point Likert scale (1: Strongly Agree; 2: Agree; 3: Neither Agree nor Disagree; 4: Disagree; 5: Strongly Disagree). Where there were multiple themes to a code, the drafted questions were based around the most recurrent themes identified from the coded data. A preamble, based on explanations in the codebook and details given in the data, was added to questions where the researchers agreed context was needed.

The draft questions were checked against a set of criteria developed by the task group to consider the inclusion/exclusion of a question; 1) Store managers and/or store owners/directors are able to speak directly to the issue; 2) The theme arises often in the Inquiry submissions; 3) The question captures the range of experiences in remote stores; 4) The question is specific enough to be able to act on/understand the issue. Questions derived from themes that had less than ten mentions (given 75% of themes had more than 10 mentions) in the data were removed from the survey, to focus on prominent themes in the anecdotal literature. Questions that enquired about similar factors under different codes were combined (e.g., store context/competition, stakeholder engagement).

#### Benchmarking for healthy stores co-design workshop

A Benchmarking for Healthy Stores Co-Design workshop was held in June 2022 in Darwin, NT, where feedback was sought from stakeholders to refine the draft survey questions. An initial recruitment email was sent to the partner organisations (*n* = 4; health organisations, *n* = 2; store management groups) inviting attendance at the workshop and input into the co-design process. Financial support for attendance of up to four key stakeholders from each organisation or communities in which the organisations operate, as well as one representative of nutrition or health from the organisation (e.g., public health nutritionist/health and nutrition manager) was offered. Partner organisations were encouraged to prioritise key Aboriginal and/or Torres Strait Islander staff/collaborators to maximise input from people residing in remote communities into the project.

This workshop covered other aspects of the Benchmarking for Healthy Stores project, as well as the environment scan component. With regards to the environment scan survey, the workshop aimed to refine the terms used to refer to a store’s health-enabling practice, prioritise environmental factors based on their impact on a store’s health-enabling practice, and determine the importance of the use of a preamble to give context to each survey question. A collaborative World Cafe approach was adopted, with small groups organised to discuss the project to share knowledge and add first-hand contextual information [34]. There were three components of the Benchmarking for Healthy Stores project discussed across three forty-five-minute sessions, including the environment scan survey tool. Participants were allocated to three tables, and facilitators for each component moved to a different table for each session.

The first group of participants were asked about what the term ‘healthy store practice’ meant to them, and responses were brainstormed. Participants were then told what the working definition of ‘healthy store practice’ was and were asked how the phrase could be changed to make the definition easier to understand. Each consecutive group added to the first group’s brainstorm.

All groups of participants were asked to prioritise environmental factors using a ‘ripple’ scoring tool, where participants ranked the environmental factors according to their impact (the bigger the impact, the larger the ‘ripple’) [35]. Environmental factors (relating to draft survey questions) to be prioritised were randomly allocated to each group, by allocating a number to each factor and using a random number generator. Participants were asked to discuss how the factor affects a remote store’s ability to increase the promotion, sale and merchandising of healthy food and drinks and limit the promotion of unhealthy food and drinks. A card listing the environmental factor was then placed on the ripple scoring tool in the middle of the table, in order to represent the group’s consensus regarding the impact the factor discussed has; the centre ripple representing ‘*little impact’*, middle ripple representing ‘*some impact’* and the outer ripple representing ‘*biggest impact’*. Key discussion points around the prioritisation of each environmental factor were recorded on a sticky note and placed onto the ripple with the card listing the environmental factor.

To test the usefulness of the preamble to the question, participants were read a question (by JB) and asked for their basic interpretation of what the question was asking, with discussion scribed by one of three researchers (EvB, MFa, JB, rotating after each group). The preamble of the question was then read out to the group, and participants were asked if their interpretation was clear, and if they found the preamble useful.

#### Development of the final survey

Scribed feedback from the workshop was collated into a word document. Questions that corresponded to an environmental factor prioritised on the ripple tool as ‘biggest impact’ were to be included in the survey. After discussion with the task group, some factors that were rated as ‘some impact’ at the workshop were included in the final survey draft, based on review of workshop notes, and taking into consideration differing opinions of store management groups versus those who worked with independent stores on the impact of the environmental factor. The survey language was then edited to improve its readability and promote ease of understanding for its intended audience, and the draft was then circulated to the task group for feedback. With feedback incorporated, the survey was converted into an electronic form using Research Electronic Data Capture (REDCap; Vanderbilt University).

#### Ethics

Ethics approval was granted by the Northern Territory Health Human Research Ethics Committee (ref: NTHREC 2021–4212) all methods were performed in accordance with relevant guidelines and regulations. Written informed consent was obtained from all workshop participants/delegates.

## Results

### Phase 1: Identification of environmental factors

One-hundred and eleven submissions were publicly available on the Inquiry website (an additional 15 submissions were listed confidentially and thus were not included in the analysis). Seventy discussion points from four of the five domains of the GFPT (*n* = 29/30 under the Strong Leaderships and Partnerships domain; *n* = 7/22 in the Community and Services domain; *n* = 9/19 in the Buildings, Public Places and Transport domain; *n* = 25/29 in the Food Businesses Domain) were included in the development of the codebook. The domain Traditional Foods and Local Food Production with its 20 discussion points was excluded due to its ongoing operation alongside the store, rather than directly influencing store operations. The final codebook was made up of 10 parent codes (as follows), with 27 child codes: Supply chain; store utilities and amenities; household utilities and amenities; store management, governance and decision-making; community structure and dynamics; store operations and practices; workforce/staffing; partnerships; healthy-eating policy and practice integration; and information systems (see Additional file 1: Supplementary Table 1).

Indexed data from the coding of the Inquiry submissions (*n* = 225 pages) produced a total of 100 themes (see Additional file 1: Supplementary Table 2), with the highest number of themes derived from the Supply Chain parent code (*n* = 25). These themes related to store health-enabling practice, including freight costs, the maintenance of transport routes, frequency of deliveries to remote areas, and the mitigating of and planning for expected (e.g., seasonal changes) and unexpected (e.g., COVID-19, cyclone) events [21]. Store governance, in reference to the organisational structures of a store and how it is controlled and operated regarding policy principles and decision-making, was another key theme identified from across the coded data, as well as how the buying power or market power of remote stores influences their ability to adopt store health-enabling practice. The submissions included both a store’s role as an essential community service, and its role as a profit-generating business [36]. Other key themes regarding consumer demand for types of food and drinks, as well as influences on community demand for different types of food and drink (such as income, household utilities and amenities, community dynamics and cultural norms) were also identified. The tension of providing affordable food alongside maintaining viable businesses was also commonly cited, with substantial coded data on the potential for price gouging and valuing profit over the health of the community, and the role of stores in fostering food security of remote communities.

Thirty-nine draft survey questions were originally developed. Eight questions were removed from the survey based on having fewer than 10 mentions in the Inquiry submissions, and one question with < 10 mentions was combined with a similar question (Additional file 1: Supplementary Table 3). These removed questions were under the following child codes: food safety; advocacy; value and support felt by staff in the workplace and community; workforce investment, training and capacity building; cost of freight; and governance/organisational structure. Five questions were merged into two as they addressed similar factors; competition and market power/alignment across stores, and collaboration and communication with industry and suppliers/community services/government and regulation. In addition to this, questions related to the child code of trading hours (*n* = 2) were removed based on their limited impact on store health-enabling practice, with the task group determining that trading hours have a more direct impact on community food security (access) [14]. This resulted in 23 draft survey questions and their corresponding environmental factors to be considered by stakeholders at the face-to-face workshop.

### Phase 2: Benchmarking for healthy stores co-design workshop

The workshop was attended by 24 delegates representing partner organisations or remote Aboriginal communities that the organisations worked within, in addition to seven study chief investigators and four project team members (total *n* = 35 attendees). Not all organisations were able to fill the four positions allocated for attendance of their delegates, and not all organisations were represented by Aboriginal and Torres Strait Islander remote community representatives. Job roles included community-based health workers (*n* = 5; both Aboriginal corporations and government) including an Aboriginal health practitioner and public health community worker. Food retail delegates also attended (*n* = 9; Aboriginal-owned and private stores) including Aboriginal store directors and chairpersons, store/area managers, retail operations and operations-support managers and representatives from merchandising. Nutritionists from partner organisations also attended (*n* = 9; including *n* = 2 from store management groups, *n* = 1 remote community Aboriginal nutrition officer). Other delegates included researchers/academics from the public health sector (*n* = 12).

When asked about the use of the term ‘healthy store practice’, the word ‘healthy’ was well-understood, with participants able to give examples that aligned with the proposed definition. The term ‘store’ was recommended to be changed to ‘in-store’ to distinguish between practices inside and outside the store. There was consensus among the groups that ‘practice’ was not a well-recognised term, with the majority suggesting the term ‘operations’, as a well-known and understood term by retailers and store owners/directors across stores. The final consensus was for use of the term ‘healthy in-store operations’.

**Fig. 2:**
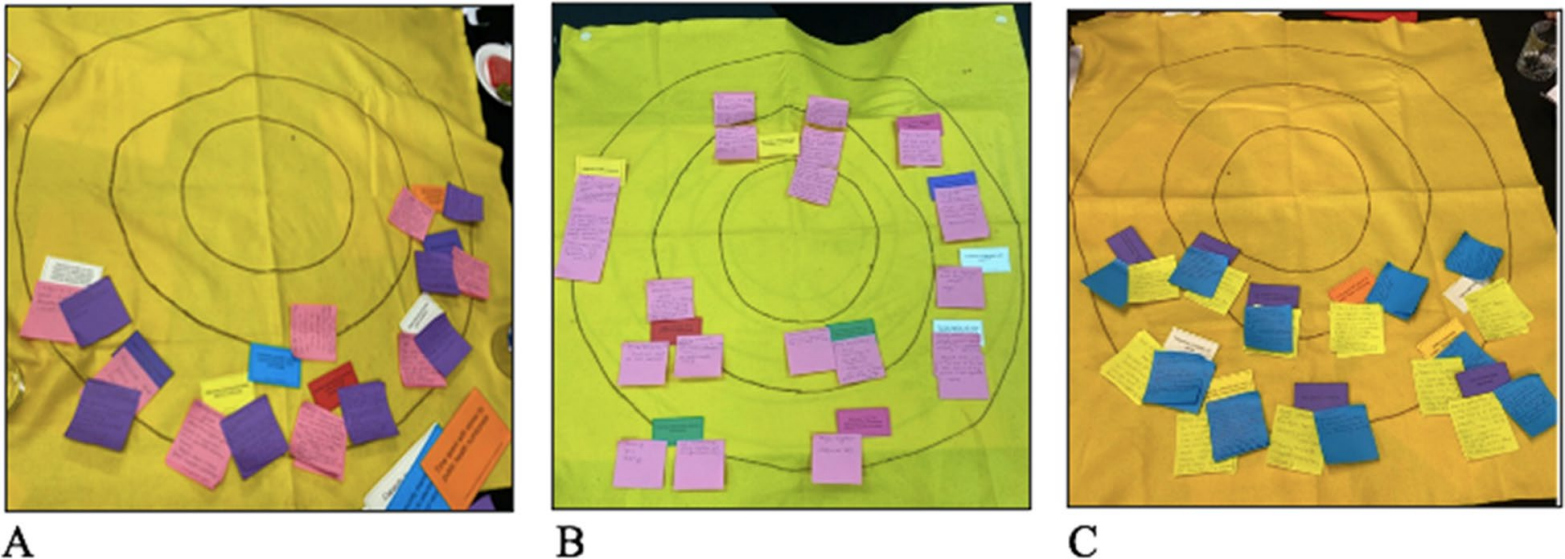
Completed ripple scoring tool

Upon completion of the prioritisation of twenty-nine environmental factors (related to the 23 draft survey questions), five factors were rated on the middle ripple as ‘*some impact’*, with all other factors being categorised on the outer ripple as ‘*biggest impact’* (see Table 2, Fig. 2). After discussion with the task group, two environmental factors (*some impact*; *n* = 2) were excluded from the survey (Store utilities and amenities (disruptions in water), Workforce stability and staffing (how long a store manager has been in community)) as the environmental factors were seen as too indirect and management values and skills were seen as more important than time spent in community (see Table 2). One environmental factor ranked as ‘*biggest impact’* (Household utilities and amenities (lack of household access to electricity for refrigeration, water, and food storage space)) was removed as store managers and/or owners would not be able to directly comment on household infrastructure as part of their role in community, resulting in 26 environmental factors included in the survey. All environmental factors related to operating costs were combined into one question, as were those about store utilities and amenities. Under the Partnerships code, time spent with stores by public health nutritionists was included after discussion with the task group to include both government and health organisation nutritionists as well as store group nutritionists.

**Table 2:** Completed ripple scoring tool

Prioritisation	Environmental Factor	Summary of Discussion
Biggest Impact	Adequate staffing capacity	Stores are unable to operate without adequate labour; Inadequate labour can mean a store has less capacity to offer healthy items (defaults to more unhealthy items, loses priority) Need to clarify what is meant by ‘adequate’: is it just having enough staff or the right kind of staff?
	Positive relationship with the community	Consensus on biggest impact
	Giving a ‘voice’ to a) community members b) store board/committee	Consensus on biggest impact
	Coming together with other stores to increase buying power	Remote stores at the end of supply chain Coming together increases visibility in supply chain, not seen if individual stores
	Capacity to negotiate with suppliers	Can combine with biggest impact of buying power
	Cost of maintenance and repairs (e.g., refrigeration)	Remote nature of stores increases costs
	Considering affordability alongside profits	The goal of the business is still viability, even though nutrition is important
	Adequate and well-functioning infrastructure (fridges, freezers)	Big contributor to operating cost, affects price and availability
	Frequency of delivery of goods	Lack of support received (through subsidisation) for more frequent delivery, lack of awareness around range of services available (rail freight or postal channels) Relief when stores have more frequent deliveries when roads become accessible following weather events
	Accessing, collecting and using data (store sales, price data)	Can be really important to stores to create competition and motivation to strive for healthier practices, differences in independent stores compared to stores belonging to a store group in their capacity to collect and use the data
	Disruptions in internet	Daily disruption to ATM, POS system, staff training and store operations more broadly
	Staffing expenses (wages, training, housing)	Large impact through wages, accommodation, limited capacity to train staff
	Cost of rent/lease agreement	Cost increasing over time; differences in payment structures across stores noted (land council, head office of retail organisations) and strategies such as negotiating long term leases
	High freight costs	Differences in impact and mitigation between store groups (impact of changes in costs are shared across store group) and independent stores (changes in freight costs are felt directly by individual store) Changes in freight can have profound effects on quality of fresh produce and perishables
	Capacity to plan for and respond to expected and unexpected events	Big impact on product availability and access
	Cost of electricity and fuel to run the store	Large impact on price, indirect impact on product availability and costs are increasing
	Effectively sharing information with the community	All practices are dependent on communication with or involvement of community
	Community members’ lack of access to household electricity, water and food storage	Primarily impacts consumer demand and community food security, stores can still enact healthy in-store operations
	Nutrition messaging of services in the community (school, clinic)	Consistency/collective impact/multi-prong strategy is key—store is more supported
	Road closures and poor road conditions	Difficulty in procuring fresh produce and other perishables that are of acceptable quality
	Disruptions in electricity	Depends on the store/context—some stores have backup generators, whereas other don’t and are greatly affected Some stores/remote communities do not have the electrical capacity to use the amount of electricity that the store needs to continue to run fridges/freezers over warmer months, resulting in ‘summer sacrificing’ – shutting down fridges
	Help for store from a. Community services b. Government c. industry/suppliers d. regulators	Support from a range of stakeholders is the most important factor for a remote store
Biggest → Some impact	Lack of healthy store practice in other stores	Varied impact identified, consensus not reached between biggest and some impact, dependent on store context
Some Impact	Community demand for different types of foods	Important but is not the biggest impact on store operations in practice
	How long a store manager has been in community	More important that the manager has the skills, is willing to engage with community and form strong relationships (even if they are new)
	Disruptions in water	Doesn’t happen very often and are warned so can plan appropriately. Some impact on takeaways, no impact on staff/other parts of the store
	Cost of repairs from break-ins (e.g., fixing a broken window)	Impact dependent on the community and frequency—can disrupt store operations but with regard to cost—most have insurance that covers this
	Time spent in stores by public health nutritionists	Independent stores rely on this more than store groups that have in-house nutritionists Depends on the type of support that is given – need celebration of enablers to healthy store environments, more meaningful relationships and strategies like recipe development
Little Impact	None

Discussion around the use of the preamble before each survey question concluded that the preamble was useful to give context to the question. Delegates also suggested there be questions included in the survey related to the frequency of which these environmental factors impact remote stores, in order to give context to answers provided.

### Final survey

A total of 20 questions (comprised of 26 environmental factors) related to perceived level of influence, and six questions related to frequency of occurrence were included in the final survey, after feedback from the task group (see Additional file 1: Supplementary Table 4). The main survey (relating to level of influence to store health-enabling practice) consisted of questions from the following parent codes; Healthy eating policy and practice integration (*n* = 2), Supply chain (*n* = 4), Partnerships (*n* = 2), Community structure and dynamics (*n* = 2), Information systems (*n* = 2), Store management, governance and decision-making (*n* = 2), Store utilities and amenities (*n* = 5), Workforce and staffing (*n* = 1).

## Discussion

This process of submission analysis, expert input and co-design with stakeholders informed the development of a 26-item environment scan survey. The submissions to the Inquiry, used in conjunction with the GFPT, provided breadth and depth of knowledge regarding the environmental factors indicated as influencing store health-enabling practice. Key themes identified as impacting store health-enabling practices mostly included supply chain factors (freight costs, transport routes, delivery, among others), weather events and seasonal changes. Store governance, the tension of achieving profits alongside healthy practices, and buying power of remote stores were also identified as prominent themes. This evidence-informed environment scan survey can be applied to remote retail settings across Australia and will provide evidence to stores and stakeholders to inform retail practice and begin to better understand the factors influencing practice and possible leverage opportunities for policy intervention.

Supply chain factors were all given high priority during the co-design workshop and were mainly described as influencing price and product availability, with varying types of transport used to ensure product availability (air, sea, rail, road, however each with different effects on product price). These themes, resulting in the development of four survey questions, focussed on the impact of supply chain factors on perishable items (fresh produce, dairy and meat products), which are often prioritised in store health-enabling practice. Internationally, maintaining supply of these types of goods has shown to be difficult due to distance to remote locations, poor temperature control, and inability to utilise cold chain transportation when using air freight, resulting in decreased quality of goods and creating uncertainty around purchasing [14, 37, 38]. In a study completed by Pollard et al., transportation issues were the most cited environmental factor by store managers in remote Western Australia, impacting on the variety and quality of perishable goods, especially fruits and vegetables [24]. Middel et al., in a review of food retail interventions in the USA indicated that transportation is a frequent issue related to the unreliability of the supply of perishable items [14].

Food affordability alongside profitability was a key theme identified from the Inquiry. The Inquiry’s focus was on food pricing and potential price gouging in remote community stores, partly accounting for the dominance of this theme. A systematic review of 41 studies, most conducted in the USA, identified that the majority of retailers had knowledge of their responsibilities for the health of their communities [14]. It showed that the strength of this influence was dependent on the strength of community relationships, and although health was considered, business viability was still of primary importance and placed at the forefront of decision-making [14]. Gravlee et al. conducted a study into understanding the context influencing store health-enabling practice in 12 communities in California and Hawaii, and found similar results, with retailers identifying sales as their top priority [38]. Loss of profit or reduced sales have been identified as barriers to store health-enabling practice, and conflicts between business interests and health interests impact the viability and sustainability of these practices [13]. However, a randomised controlled trial conducted in remote Australia gave world-first empirical evidence that store health-enabling practice does not always equate to a loss in profits [11]. It also found that study success hinged on the partner Aboriginal Corporation prioritising the health of the communities they served alongside the organisation goal of operating viable remote store businesses [32]. The Australian remote store setting, especially in the NT, has some supports in place such as nutrition policies and Community Stores Licensing (under the Stronger Futures in the Northern Territory Act 2012, ended in mid-2022 and continued through the NT Food Security Program) to ensure nutrition and food security are considered during decision-making processes [39–41].

The buying power or market power of remote stores (i.e., ability to influence prices through manipulating supply/demand of a product), impacts resources and supply availability. Store management groups have been established, to utilise the market advantages to influence price [42–44]. However, even with the development of store management groups, a multitude of remote stores still only represents a small fraction of the buying power of a major supermarket in Australia [22]. Remote stores are limited by buying power, facing higher wholesale prices compared to larger supermarket chains, with some stores paying retail prices for goods due to utilising major supermarkets as stockists [22]. This supports the inclusion of survey questions relating to both buying power and storage facilities, so as to capture the nuances of the remote store context. International evidence suggests that supply chains are not designed to best serve minority or remote areas, with limited ability of suppliers to be able to focus on specific communities, a finding which holds relevance for the remote Australian context. Lack of contractual obligations with suppliers, which can occur between suppliers and smaller remote retailers, can increase price volatility [37, 38]. A systematic review of reviews by Gupta et al., showed that increased power or market control can be both a barrier and enabler to healthy store practice, exacerbated by power imbalances between retailers and suppliers [13]. Stores that have more market control are likely to tend to favour profit over health but have the capacity to access the space and resources to implement and support healthy retail initiatives.

Community demand and its influences, including income, household utilities and amenities, community dynamics and cultural norms were identified as prominent themes impacting on healthy-in-store practices. These factors align with a recent systematic literature review showing consumer needs and preferences can work as both a barrier and an enabler to store health-enabling practice [13]. However, feedback from key stakeholders at the workshop indicated consumer demands for different foods have *some impact* on store health-enabling practice, with delegates stating it as an important factor, but not as the *biggest impact* on store practice. Household utilities and amenities (the access to electricity, water and food storage facilities), potentially impacting the demand for different foods, was given high priority during the workshop, however, this was not included in the final survey as the intended survey participants (remote retailers) would not be able to directly comment on household infrastructure as part of their role. In addition, support and partnerships with public health nutritionists (including government, health and store group nutritionists) was seen to have *some impact* on health-enabling practice. However, aligning with the literature, with stakeholder partnerships identified as an important enabling factor in creating healthy retail food environments [13], the decision was made to include this environment factor.

The rigour of this survey tool was strengthened by drawing upon the co-designed and evidence-informed GFPT to identify preliminary environmental factors relevant to the remote retail food environment [19]. Sampling bias may have influenced the study outcome as the primary data used to produce the survey (the Inquiry submissions) were obtained through convenience sampling. However, many key organisations related to remote food retail in Australia made submissions to the Inquiry, indicating the perspectives of these stakeholders were incorporated into the analysis. Our derivation of themes and their prioritisation from this dataset was informed by experts with extensive knowledge of the remote food retail context. Our method also assumed all Inquiry submissions were relevant to the remote community context, though factors that had < 10 mentions were excluded from the data set to ensure we identified factors common across contexts, which has potentially left out important information applicable to some communities. However, our method ensured the capture of prominent themes and those considered by experts to have important impact on remote store operations. While the Inquiry focussed on pricing only, one of four store health-enabling practices, the determinants of pricing also impact other store practices, highlighting the systems-effect of remote food retail. In addition, the co-design approach for the development of the tool has produced a robust survey that is relevant to the end user. This survey has the potential to give an updated examination of factors that affect the retail food environment in a remote setting, with a renewed focus on the healthiness of the in-store environment. It will also give the opportunity to compare between stores and communities, enabling a nuanced understanding of the identified environmental factors in the remote setting.

Among the team of authors (1 Indigenous, 9 non-Indigenous), five have lived in the remote setting and one resides in a remote Aboriginal community. Nearly all authors have extensive knowledge and/or experience regarding both Aboriginal and Torres Strait Islander communities and the remote retail sector and several have spent long periods of time in remote communities over many years. The authors share the belief that having consistent access to sufficient nutritious, culturally appropriate, good quality food at an affordable price is a human right and one that must be advocated for to achieve social justice and equity. Across the duration of the study, the researchers discussed the lens through which they viewed the data, in order to mitigate bias that arose from their own values.

## Conclusions

This study provides a rigorous, stepped methodology in the co-design of a survey tool to examine environmental factors affecting remote store practice, through the review of submissions to a Australian Parliamentary Inquiry informed by a pre-existing remote Aboriginal and Torres Strait Islander food systems planning tool and extensive consultation and workshopping of the survey tool with stakeholders. Environmental factors the survey examines, including but not limited to supply chain (road conditions, the cost and frequency of freight), governance of stores, store relationships with community, and maintenance of infrastructure, and their impacts on food pricing and health promoting initiatives especially, have been brought to the attention of policy makers for decades. In Australia, governments have demonstrated concern around these impacts, through inquiries into remote food pricing and food security, as well as the annual assessment of food pricing through the NT Government Market Basket Survey. However, there is yet to be a systematic investigation of the extent of these factors and associated impact on remote store operations. The wide application of this tool, that has been co-designed with those experiencing first-hand the impacts of these factors across remote Australia, has the ability to generate powerful data for policy makers at the local, state and national level to enable healthy retail food environments, and has the potential for producing evidence for remote contexts internationally. The survey tool could also be useful for other contexts, particularly remote settings such as those in Canada and other parts of North America. The development and circulation of this survey tool is timely in Australia, given the current development of a National Remote Food Security Strategy, and globally, with the progressive adverse impacts on food supply and access that the world is facing with human-induced climate change. The data created through the use of this tool will be able to be fed back to stores for their use to inform and improve store policy and apply for funding at the local level to mitigate the impact of these factors. Use of the environment scan survey in remote contexts in Australia and internationally has potential to capture the diversity of store context and provide evidence to create remote store environments that are conducive to community health and wellbeing.

### Supplementary Information

**Additional file 1: Supplementary Table 1.** Codebook. **Supplementary Table 2.** Summary of themes extracted from the Parliamentary Inquiry into food pricing and food security in remote communities. **Supplementary Table 3.** Worked question matrix. **Supplementary Table 4.** Survey tool.

## Data Availability

The datasets used and/or analysed during the current study are available from the corresponding author on reasonable request.

## Abbreviations

CAQDAS: Computer Assisted Qualitative Data Analysis Software
DALY: Disability-adjusted life years
GFPT: Good Food Planning Tool
NT: Northern Territory
REDCap: Research Electronic Data Capture
USA: United States of America
