# Supplementary material for: Development of a survey tool to assess the environmental determinants of health-enabling food retail practice in Aboriginal and Torres Strait Islander communities of remote Australia
Source: BMC Public Health. 2024 Feb 12;24:442. doi: 10.1186/s12889-024-17945-9 (PMC10863203; doi:10.1186/s12889-024-17945-9)
Supplement: Supplementary file 1 — Additional file 1: Supplementary Table 1. Codebook. Supplementary Table 2. Summary of themes extracted from the Parliamentary Inquiry into food pricing and food security in remote communities. Supplementary Table 3. Worked question matrix. Supplementary Table 4. Survey tool. [file 12889_2024_17945_MOESM1_ESM.pdf]

SUPPLEMENTARY MATERIAL

Contents

Supplementary Table 1: Codebook 1

Supplementary Table 2: Summary of themes extracted from the Parliamentary Inquiry into food pricing and food security in remote communities 9

Supplementary Table 3: Worked question matrix........ 37

Supplementary Table 4: Survey Tool........ 42

Supplementary Table 1: Codebook

Parent Code	Sub/ Child code	Description of code	Data to exclude
Supply chain	Cost of freight	This code refers to freight costs influencing remote store policy and practice	
	Maintenance and functioning of transport and delivery	This code refers to supply chain factors (food availability, transport and delivery, and contracts) which could influence store policy and practices either positively or negatively. It also encompasses the storage of foods during transport and the condition the food arrives to stores in.<br><br><u>Examples:</u><br>• Good availability of refrigerated trucks & appropriate food safety procedures ensuring that produce arrives in excellent condition<br>• COVID-19 disruptions to supply chain (food availability as well as border closures)<br>• Poor maintenance of freight routes precluding food supplies from being delivered to remote stores	
	Industry and supplier relationships and support services	This code refers to relationships between stores and suppliers or manufacturers, including manufacturing products to suit remote store goals, and the ability to negotiate or access support services.<br><br><u>Examples:</u><br>• Stores using local suppliers where possible<br>• Supplier rebates being accessed by stores	
	Supply chain planning & risk mitigation	This code encompasses any measures taken or not taken to plan for wet season and other (expected and unexpected) events, as well as whether strategies to optimise the cost of delivery and transport have been considered.<br><br><u>Examples:</u><br>• Storing extra foods on-site to prepare for wet seasons disruptions<br>• A remote community store has limited stock during the wet season due to a lack of	

		planning for suitable transport for the delivery of foods to the community (barrier)<br>• Store and community services (aged care, school, health service) combine orders and deliveries to minimise transport costs where possible	
	Store context (community and store size, business competition) influencing supply chain dynamics<br>*Note: this was not included in GFPT, but added after consultation with Task Group*	This code refers to community or store size, as well as the surrounding business/ retail competition, proximity to other food retail businesses and having the capacity to influence merchandising policy or practice in terms of stock order quantities, buying power based on differences in consumer demand.<br>Example:<br>• Smaller stores not being able to buy in volumes that are perhaps as economical as larger stores which could make it difficult for smaller stores to stock a large range of products or offer promotions on healthy products compared to large stores	
Utilities and Amenities (store)	Physical access to functioning utilities and amenities (store)	This code relates to the degree to which stores have consistent access to adequate utilities and amenities in, including electricity, water, sufficient and functioning facilities for food storage (including refrigeration), preparation, sale and consumption (including safe transport from store to homes). It also covers the condition and maintenance abovementioned utilities and amenities to ensure they are in working order. Food hygiene facilities are also encompassed here (sinks, cleaning supplies, bathrooms etc).<br>Examples:<br>• Stores have consistent access to refrigeration and freezers which permits a wider range of foods to be stocked<br>• The store struggles to maintain the quality of fruits and vegetables without a consistent supply of electricity (barrier)<br>• All stores have adequate and clean water to carry out hygiene practices in line with food safety guidelines	
	Economic access to utilities and amenities (store)	This code refers to the retail context. Refers to whether stores have access to financial resources required to use utilities and amenities (e.g. air conditioning, refrigeration, cleaning supplies) and pay for their maintenance/ upkeep. This also encompasses the costs of leasing or owning the store premises.<br>Examples:<br>• Food in store spoiling due to inadequate refrigeration based on financial constraints.	
Utilities and Amenities (households)	Physical access to functioning utilities and amenities (households)	This code relates to the degree to which homes have consistent access to adequate utilities and amenities, including electricity, water, sufficient and functioning facilities for food storage (including refrigeration), preparation and consumption. It also covers the condition and	

		maintenance of above mentioned utilities and amenities and whether they are in working order. Food hygiene facilities are also encompassed here (sinks, cleaning supplies, bathrooms etc).<br><br><u>Example:</u><br><br>• Homes have relevant health hardware required for preparation and storage of food e.g. fridge, above-ground storage.<br>• There is no clean water supply to households in the community (barrier)	
	Economic access to utilities and amenities (households)	This code refers to the household context. Refers to whether homes have access to the financial resources required to use utilities and amenities (e.g. air conditioning, refrigeration, cleaning supplies) and pay for their maintenance/ upkeep.<br><br>Example:<br><br>• Households are not purchasing frozen foods as they cannot afford a functioning freezer (barrier)	
Store management, governance and decision making	Management values and leadership	This code refers to the values guiding management of the store, which could be either a barrier or enabler of healthy merchandising policies and practices. This includes the degree to which managers and/ or store directors/ owners understand their legal duties and requirements and are willing to make decisions to benefit the community. This encompasses individuals' values/beliefs/perspectives as well as whether business plans' consider health promotion principles alongside economic objectives. The code also includes whether managers/leaders are acting as role models for other community members, and the level of support or promotion of Indigenous management.<br><br><u>Examples</u><br><br>• The store manager, store owner or store director commits to subsidise fruits and vegetables to promote healthy eating within the community, despite the cost.<br>• The store is hesitant to implement interventions that will alter profits of the store (barrier)<br>• Management values align with the values of the organisation governing the store	Exclude data describing *who* is in management
	Governance/ organisational structures	This refers to remote stores' governance/ management/ organisational structures comprising individuals with relevant knowledge and skills (including Indigenous community members). It also refers to the stability of store boards/ governance/management, and how they are recognised by the community.<br><br><u>Example:</u><br><br>• Store board is made of community members who represent the needs and values of the community	Exclude values of those in governance structures

		• Store board members have been involved for a period of time which allows for consistency and stability within governance and management<br>• Store management is unstable, with frequent changes in ownership and management hindering implementation of healthy merchandising policies and practices	
	Policy-making and decision-making processes	Refers to the logistic (adequate meeting places, regularity of meetings) and social aspects (respect for others' views) of decision-making in the store, and whether these factors are barriers or facilitators of healthy food retail policy and practice. The code also encompasses whether committee and community consultation and engagement is built into store policy and decision-making processes, and whether committee and community are able to effectively influence store rules and practices.<br><br><u>Examples:</u><br><br>• Store committees meet once every three months<br><br>• Community members/customers are consulted and engaged throughout the policy or decision-making process to ensure community acceptance of store merchandising practices.<br><br>• Despite there being opportunities for community engagement and consultation, community feedback is not effectively incorporated into decision making regarding store policy and practice	
Community structure and dynamics	Community harmony, pride in and support of the store (and staff/ management)	This relates to external community factors or dynamics that influence store policy and practice, either positively or negatively. It acknowledges that the degree of community support of and pride in the store may influence retailers in their merchandising practices, which could either enhance or hinder healthy merchandising practices. Broader community harmony incorporates relationships between families, services and community members that could promote or hinder the store's ability to implement healthy retail policies and practices.<br><br><u>Examples:</u><br><br>• Community members (including Elders) respect the store manager and support store policies and practices aiming to improve the health of the community<br><br>• Unrest within the community leading to the store being broken in to which impacts implementation of healthy merchandising practices.	
	Community motivation and demand for healthy or unhealthy foods	This code acknowledges that community food preferences and demand for different products is likely to influence retailer's choices in product availability and promotion. This could either promote or hinder healthy food retail environments, depending on the preferences of	

		the community. For example, if the community indicates a high demand for discretionary items, this may lead a retailer to be hesitant to implement merchandising practices to dissuade customers from purchasing these items. Conversely, community members indicating a preference for healthy food items could give a retailer confidence to stock a wider range of healthy products.<br><br><u>Example:</u><br>• Healthy cooking program at the local school influences kids and their families to seek healthier food options, creating a higher demand for certain products<br>• Remote store managers noting that the large sizes of hot chips, pies and soft drinks are generating significant sales (indicating community preference), so may be unwilling to consider decreasing portion sizes or modifying product availability to promote healthier choices in the community<br>• Store manager's claiming that healthy foods don't sell, assuming this to be indicative of low demand and therefore being reluctant to implement healthy food retail policy/ practice in fear of loss of sales	
Store operations and practices	Trading hours	The extent to which food businesses are open during hours that meet the needs of the community.<br><br><u>Example:</u><br>• Store not being able to open due to community events (e.g. funerals with multiple day duration) or weather events (flooding, no electricity)	
	Food safety practices	The extent to which food safety guidelines are in place and work well (such as adequate stock rotation, safe storage, no out of date food, no contamination from pests, no dogs or animals permitted on premises)<br><br><u>Example:</u><br>• Meat is stored away from fruit and vegetables, and is kept in refrigerators at the required temperature<br>• Food on the shelves is out of date due to inadequate stock rotation and auditing processes	
Workforce/ Staff	Workforce investment, training and capacity building/ skill development	This code refers to whether there is support and investment in building job skills in food and nutrition to strengthen workforce capacity and develop leadership skills of all staff.<br><br><u>Examples:</u><br>• There is regular staff training on store nutrition policies and practices<br>• Investing time and funding towards staff training and skill development is valued by management to strengthen workforce capacity	

		• Staff lacking the skills and confidence to implement healthy merchandising policies and practices due to inadequate training programs	
	Workforce stability and staffing<br>*Note: this was not included in GFPT*	This code refers to changes in workforce stability and staffing factors which have the capacity to either positively or negatively influence store policy and practices, including trading hours as well as the capacity to implement healthy merchandising policies and practices.<br>Examples:<br>• Staff impacted by COVID-19 (either being unwell or in isolation) impacting a store's trading hours<br>• Staff needing to attend family or community events, meaning they are not available to work	
	Value and support felt by staff in the workplace and community	This code refers to the extent to which food retail staff, store board, nutritionist are valued within the community and whether they are supported to take action in the remote food retail setting. The degree to which food retail workplaces (and workplaces of other key stakeholders) provide a safe and respectful environment is also encompassed within this code.<br>Example:<br>• Staff opinions are valued in the workplace and taken into consideration when making decisions.<br>• Harassment within the food retail workplace leads to staff members feeling unsafe and unsupported	
	Staff/ retailer support of store	The degree to which food retail staff (include store board, managers, retail staff and nutritionist) are willing to take action for the good of the community.<br>Example:<br>• Staff are motivated to implement healthy merchandising practices as they understand and/or observe the potential benefits to community health and wellbeing.<br>• Storeowners or managers do not perceive themselves as having a responsibility to consider the communities' health within their actions and decision making at the store level	
	Cost of staffing	This code refers to the costs of staffing in remote communities having an influence on the capacity of the store to implement best-practice healthy merchandising policies and practices.<br>Example:<br>• Relocation costs required to fill staffing positions in remote community stores<br>• Difficulties with staff retention associated with higher costs for recruitment and training as well as	

		abovementioned relocation costs where necessary	
Partnerships	Stakeholder communication and collaboration	This code relates to whether stakeholders create effective partnerships through communication and collaboration. It involves the extent to which all relevant stakeholders (nutritionists, govt and non-govt organisations, researchers, universities) actively participate in improving the remote food retail environment.<br><br>Collaboration refers to the degree to which stakeholders work on joint projects and share resources amongst stakeholders to ensure a multi-sector and multi-disciplinary approach that meets community needs.<br><br><u>Examples:</u><br>• Nutritionist works with food retail stores to regularly assess food quality and compliance with nutrition guidelines<br>• University student placements in conjunction with remote stores<br>• Poor communication between stakeholders delays the progress of projects and policy development that could improve the remote food retail environment	Note: 'resources' here does not refer to information as this is covered under 'information systems' below
	External partner responsiveness to community needs	The degree to which external services (such as researchers, health professionals, government officers) are responsive to community needs and support action plans already in place<br><br><u>Examples:</u><br>• Monash University collaborating with community members to guide research agenda that is co-designed and meets community needs<br>• External partners not being flexible and adaptable to community needs and autonomy within project partnerships	
Healthy-eating policy and practice integration	Alignment of vision, goals, policies, plans and practices/ actions related to healthy eating	This code relates to the degree of consistency and stability in vision, goals, policies, plans as well as in practices/ actions relating to healthy eating across the community, as well as between communities. This encompasses whether community plans and policies (not just store-related policy/plans) consider affordability and quality of food for the whole community, both in the short and long-term. It acknowledges that if policies, plans or practices relating to nutrition vary across the community, it could make it more challenging for retailers to implement healthy merchandising practices/ policies. It also acknowledges that store policies, plans, practices and contracts may be influenced by national, state and local guidelines/ policies, which could either positively or negatively influence the store's implementation of healthy food retail policies and practices. This code also refers to the extent to which nutrition-related goals, plans and actions are monitored and reviewed regularly to ensure ongoing policy integration and alignment across the community.<br><br><u>Example:</u>	

		• School healthy lunch program providing discretionary foods in contradiction with remote food retail merchandising practices dissuading discretionary food purchase. This could impact community demand and support for the store's attempt to promote healthier purchasing behaviours.<br><br>• Community engagement events holding sausage sizzles organized by various stakeholders where only sausages, white bread and tomato sauce is served.	
	Advocacy	This code refers to whether the store is able to engage in advocacy (either alone or alongside partners) if a particular action is outside the remote store's ability.<br><br><u>Examples:</u><br><br>• Remote store manager advocating for support from health service or even store board to implement healthy merchandising practices<br>• Remote stores partnering with other community services to publish submissions for policy changes that would impact remote store nutrition policy/ practice<br>• Retailers/ store managers/ owners lacking required skills and the network necessary to advocate for policy changes that they feel are necessary to improve the remote food retail environment to benefit the community.	
Information systems	Information collection, analysis and application	This code refers to whether store data (or other relevant data sources) is routinely collected and analysed, and whether this information is applied to inform decision making to alter store policy and practice.<br><br><u>Example:</u><br><br>• Collecting store sales data to influence ongoing promotion of healthy food that could have a direct influence on community dietary intake<br><br>• Store sales data is infrequently collected due to time constraints, meaning the store owner is not able to get an accurate picture of the impact of healthy merchandising practices on business and community health outcomes.	
	Information sharing and dissemination	This code refers to the extent to which nutrition and store-related information (including policies and plans) are accessed by community members and shared with stakeholders. It also encompasses whether positive changes (within the store) are widely shared and celebrated with the community. It acknowledges that the degree of information sharing and dissemination could influence community perceptions of	

		transparency among food retail businesses. This, in turn, could influence the extent to which community members and services support the store as well as the motivation of retailers to maintain healthy merchandising practices.<br><br>Example:<br><br>• Results of Healthy Stores 2020 easily accessed and celebrated by the stores and communities involved.<br>• Retailers freely sharing the store sales data with stakeholders which could then influence policy and practice through process evaluation.<br>• Community members lose trust in the store owners and managers since retail information is not openly shared.	

Supplementary Table 2: Summary of themes extracted from the Parliamentary Inquiry into food pricing and food security in remote communities

**Parent Code**			
	**Child Code**	**Themes**	**Subthemes**
**Healthy Eating Policy and Practice Integration**			
	Advocacy	Stakeholders coming together to advocate (+ve)	Different stakeholders (stores, store groups, researchers, health councils, Aboriginal organisations) coming together to increase power of advocacy (to manufacturers, government)
		Lack of skills/resources required to advocate (-ve)	Stores not having the skills or resources to apply for funding or grants
	Alignment of vision, goals, policies, plans and practices/actions related to healthy eating	Alignment across stores	Inconsistencies in price displays making it difficult for community members to budget or choose products effectively
			Differences in nutrition policies/practices across stores in a community hinders their ability to implement nutrition interventions (may result in loss of profit)
		Stores Licensing Regime	Inconsistent monitoring and assessment of stores licensing across stores resulting in inconsistent nutrition practices. Repercussions for non-compliance are varied.
			Store licensing regime is unclear or difficult to understand

Parent Code			
	**Child Code**	**Themes**	**Subthemes**
			Stores licensing regime only exists in one jurisdiction (NT); helping to bring stores up to the same benchmark nutrition standard across licensed stores, with other jurisdictions lacking a comprehensive monitoring system
		Alignment of services within the community, between communities and across jurisdictions	Misalignment of nutrition messaging between services within the community hinders the sustainability of interventions (e.g. school messaging or clinic messaging does not align with what foods the store has available; restrictions on discretionary foods sold at the store but vending machines available at the police station)
			Services within the community work together to implement nutrition interventions/practices/messaging across services
		Policy environment	Misalignment of policies across government portfolios for remote communities hindering the implementation of nutrition interventions in communities (e.g. the keycard or income support hindering the investment in selling locally produced food)
			No sustainable/consistent funding stream for a national nutrition policy (governmental) for remote communities
			Differences in the baseline/minimum terms of compliance between policies in remote communities (lease agreements by Land Trusts and stores licensing agreements having different nutrition minimum standards)
**Store Operations and Practices**			
	Food safety practices	Level of adherence to selling foods only fit for consumption (contamination) (n=11)	Contamination (from pests, mould)
			Selling foods past their used by dates (including thawing and refreezing products)
			Inappropriate labelling/packaging (no nutritional information,

Parent Code			
	**Child Code**	**Themes**	**Subthemes**
			products decanted into zip-lock bags)
		Storage of food impacting on ability to stock fresh produce/ purchasing of foods (n=2)	temperature control challenges
			inappropriate storage conditions (foods stacked on top of each other in fridges, dirty shelving)
		Resources (financial, labour, knowledge) allocated to food safety (n=4)	Costs of adhering to food safety (throwing out foods that are unfit for consumption resulting in waste and loss of revenue)
			lack of staff food safety knowledge
			Resources for staff training on food safety (large store groups having more resources to allocate towards training)
		Food safety regulation (n=2)	Poor monitoring and enforcement of regulations
			Regulations altering order practices (less fresh produce) to minimise losses (impacting on supply of fresh produce in the store, -ve)
	Trading Hours	Community consultation and responsiveness to community needs	Consulting community as to what their needs are in regard to trading hours of the store (taking into account income streams)
			Closing during sorry business, community meetings as a sign of respect for the community
		Variability of trading hours between stores and between communities	Extended trading hours/differing trading hours of competitor stores (impacting on ability to implement nutritional policy or misalignment of nutritional goals- increased purchases of discretionary foods from competitors)
		Impact of community unrest on trading hours	Managers use closing the store as disciplinary action against the community (-ve)
			Break-ins impact ability for store to open, staff feel too unsafe for store to open
		Store resources, utilities and amenities impacting trading	Inadequate staffing (-ve) is a barrier to opening the store

**Parent Code**			
	**Child Code**	**Themes**	**Subthemes**
		hours	Stocktake results in a store closing or closing early (neutral, normal practice)
			Inability to access functioning store utilities results in store closure (e.g., electricity)
**Information Systems**			
	Information collection, analysis and application	Food Basket data/affordability of food	Voluntary collection/participation of stores creates incomplete data and may skew data to stores with lower prices
			Collected data does not include all stores (does not include the most vulnerable stores e.g., those with the highest freight costs) which compromises comparison ability
			Lack of data collection/surveillance in some jurisdictions due to lack of funding
		Resources/tools to collect data	Lack of training for data collectors can result in errors or incomplete data
			University students (nutrition and dietetics) aid in data collection, reducing the resources required from stores
			Researchers developing tools (web-based) to aid in data collection at the store level
			Complexities in collating the data, resulting in data not being used
		Data collection/surveillance	Lack of evaluation of practices/policies (stores licensing regime, national nutrition education program) results in difficulties applying the data for improvement of policies
			Lack of monitoring/monitoring stores licensing compliance based on risk can result in changes to food security in a community being unidentified
			Use of different survey tools/inconsistent methodologies/lack of routine collection across jurisdictions makes comparison difficult

Parent Code			
	**Child Code**	**Themes**	**Subthemes**
			Due to lack of data collection, only anecdotal data is available to guide practice in some jurisdictions
			There is no avenue for complaints in some remote communities, with surveillance only if authorities are alerted to an issue
		Use of data to guide practice	Stores (mostly store groups) using data to guide solutions to pricing issues, store sales data to guide nutrition interventions (monitor profits etc.) and policies and feedback to guide capacity building
	Information sharing and dissemination	Transparency of policies, governance and management practices	Publically available policies (nutrition, pricing, decision-making, government) and data (sales, pricing) increase the trust of the community and help hold accountability and monitor compliance
			Lack of transparency of data and governance (e.g., sales, data, use of profits) to community and store boards impacts negatively on policy and decision-making
			Lack of publicly available or wholly-available (to every service that requires the information) data (pricing, policy, sales) hinders monitoring of compliance or opportunities to improve practices
		Presentation of data during dissemination (e.g., anonymised, incomplete, consolidated)	Anonymised data or incomplete data creates difficulties in drawing comparisons between stores and jurisdictions
			No consolidation of data to be able to inform progress or reiteration of practices/policies
		Sharing of information between stakeholders to help or hinder partnerships	Sharing of information and feedback with community members can help to facilitate demand for healthier options
			Sharing of information between different service providers in the community (e.g., health, clinic, police)
			Lack of transparency around the governance of the store prevents potential partnerships (with store

Parent Code			
	**Child Code**	**Themes**	**Subthemes**
			managers appearing the only point of contact)
**Community Structure and Dynamics**			
	Community harmony, pride in and support of the store (and staff/management)	Community unrest impacting store practice	Stores closing as a punitive measure or due to theft, pilfering or staff feeling unsafe (as well as associated additional costs)
			Community unrest impacting freight or transport of goods (e.g. rocks being thrown at trucks)
			Increased demand for the store with an influx of people into community for cultural events such as funerals
			Community violence impacting Indigneous employment at the store
		Store practice influencing community support for the store	Higher prices and lack of subsidised freight impacting negatively on community support for the store
			Store is seen as the 'hub' of the community, increasing support for the store and store projects
			Community support for store management groups to manage the community store/ community control of profits increases pride and support of the store
			Lack of transparency, frequent turnover of management and lack of community trust decreases support for the store
			Store servicing homelands increases support of the store (catering to all community members)
		Family/cultural dynamics	Community members working at the store can influence who can shop at the store and when they can enter the store (e.g. customer cannot shop at the store when 'poison cousin' is working)
			Community members view the store and the ready availability of 'western' food as a reason traditional food knowledge has been diminishing

**Parent Code**			
	**Child Code**	**Themes**	**Subthemes**
	Community motivation and demand for healthy or unhealthy foods	Income of community members affecting motivation and demand for healthy food	Decreased income (due to fortnightly pay cycles, CDP financial penalties, BasicsCard restricting finances to the store) results in reduced demand for higher priced items and increased demand for energy-dense, nutrient-poor products
			Increased income support as a result of Covid-19 increased demand from the store (including demand for fresh fruits and vegetables)
		Movement of people in and out of community	People coming into community for cultural/family reasons (e.g. funeral) or as a result of the Covid-19 pandemic response, increases demand from the store, putting pressure on supply chains
		Availability of food elsewhere other than at the remote store	Community members who have access to transport have the ability to shop in nearby towns (cheaper prices and more availability)
			The degree of hunting, gathering and other traditional food procurement in a community affects the demand for goods at the store
		Influence of community access to functioning utilities and amenities on motivation and demand	Lack of access to potable water in community increases demand for soft drinks at the store
			Lack of community access to functioning kitchen infrastructure to store, prepare and cook foods increases demand for convenience, ready-to-eat foods as well as increased frequency of purchasing (purchasing from the store at least once a day)
		Store practice influencing demand for certain products	Increased demand for energy-dense, nutrient poor foods due to marketing and merchandising in the store
			Store not responding to community needs with bulk-buy packages available instead of convenient serve sizes of fresh fruits and vegetables
			Low quality of fresh fruits and vegetables (long travel times to get to store) and their short shelf

Parent Code			
	**Child Code**	**Themes**	**Subthemes**
			life reduce demand/motivation to purchase
			Nutrition marketing delivering the same message from stores across other services in the community (e.g. school, clinic) increases motivation for healthy eating/purchasing practices (+ve)
			Smaller populations have increased ability to affect what is available in store through their demand for products
			Low elasticity of supply and demand in remote stores (no change in demand in reaction to price changes)
		Community dynamics/cultural norms and practices	Lack of nutrition education/skills/health literacy (and limited opportunity to gain these skills) decreases demand for healthy items
			Community brand loyalty, and rations of flour, tea and sugar as a result of European settlement, impact what is purchased from the store and the community's ability to adapt to changes in stock
			Cultural norms of sharing food with family increases demand for convenience, ready-to-eat foods and decreases motivation for healthy, prepared meals (due to inability to store large amounts of food for extended periods of time)
**Household Utilities and Amenities**			
	Economic access to utilities and amenities (households)	Inconsistent access and ability to pay for electricity and running water	Power cards running out of pre-paid funds which can also contribute to overcrowding
			Blackouts leading to loss of food
			Inability to afford electricity and water encourages reliance on ready made meals; high costs constraining food budgets
			Utilities cost contributing to food insecurity in remote communities
		High cost of housing and cooking equipment	Lack of storage capacity in homes increasing demand for

Parent Code			
	**Child Code**	**Themes**	**Subthemes**
		impacting capacity to purchase healthy food	ready to eat foods
			High household repair costs increasing reliance on ready to eat foods
			Proportion of income on housing impacting budget for healthy food
			Stores trying to promote affordability of whitegoods by not passing on freight costs
			High cost of refrigeration and leasing arrangements for whitegoods
	Physical access to utilities and amenities (households)	Reliance on ready-to-eat, convenience meals	Non-perishable, processed foods are easier to store
			Inappropriately/inadequately equipped kitchen facilities or functioning kitchen facilities reduce ability to store, prepare and cook foods
		Increased food waste or food not fit for consumption	Unreliable power supply with regular disruptions and the use of pre-paid power cards result in increased food wastage or spoilage
			Local climate, including high humidity and extreme weather events, with houses not built for purpose, increasing food spoilage
			Overcrowding in remote community housing decreasing the ability for good food hygiene
			Limited access to free, filtered, clean, good quality water
		Barriers to the ability to store, prepare and cook food, impacting on food security	Cultural obligations of sharing of food and housing leading to limited opportunity to store food to cook meals
			Poor initial construction of housing and health hardware, overuse (due to overcrowding) and lack of regular maintenance and poor functionality a barrier to safe storage, preparation and cooking of food
			Limited ability to stockpile food during adverse weather events (e.g., cyclone season) due to lack

Parent Code			
	**Child Code**	**Themes**	**Subthemes**
			of storage
			Decreased access to whitegoods due to high freight costs for store
**Partnerships**			
	Stakeholder communication and collaboration	Public health nutrition workforce	In-house nutritionists for large store groups supporting stores to implement healthy store practices
			Public health nutrition workforce in/working with remote communities lacking capacity to work effectively with remote stores
			Public health nutritionists having no involvement with remote stores or there are significant barriers to working with stores, inhibiting their ability to implement healthy practices
		Stores working with researchers	Researchers/universities working with remote community stores to design, implement and evaluate in-store health strategies with the aim to improve nutritional outcomes
			Large store groups being open to/having strong relationships with researchers and universities - increasing resources available to stores to implement healthy practices into store operations
		Collaboration/communication with industry and suppliers to ensure supply	Catalyst of Covid-19 bringing together stakeholders/fostering collaboration between, essentially, competition (major supermarkets and suppliers) in order to ensure supply to remote communities
		Extent of collaboration with community/services within the community	Collaborating with services in the community (police, health centre)
			No consultation with community when designing and implementing strategies
			Store groups not collaborating effectively with community
		Government collaboration and action	Teams across sectors of government working together to have a multidisciplinary approach to food security in remote

Parent Code			
	**Child Code**	**Themes**	**Subthemes**
			communities
			Formation of coalitions/task forces in order to aid in communication/advocacy to government
			Inaction of government despite extensive communication/research of recommendations/what needs to occur
	External partner responsiveness to community needs	Responsiveness of the government (federal, state, territory) to community needs	Inaction of the government despite having the information around what communities need
			Government providing funding to initiatives that fail to deliver intended outcomes
			Disconnect of states participating in interventions resulting in failure to sustain the intervention
		Inadequate, limited or inequitable funding of stores and programs	Funding of stores not aligning to their market share (store groups receiving more funding)
			Limited or short term funding for programs in remote stores
		Regulation and continued monitoring	Response to community complaints or concerns - inadequate investigation of community complaints by relevant authorities resulting in community feeling they are being ignored
			The use of stores licensing to regulate store health policy
			Distance of remote communities and time taken to travel hindering the ongoing monitoring of stores
		Culturally appropriate, community-led interventions	Westernised delivery of services not suitable for remote community members
			Interventions included community consultations during the design phases, resulting in good behaviour change
			Lack of understanding of community needs and no consultation leading to unsuccessful interventions based on assumptions made

**Parent Code**			
	**Child Code**	**Themes**	**Subthemes**
		Support of decision-makers (store, store boards) by other stakeholders	Increased resources resulting in better outcomes (e.g., researchers supporting stores to implement changes, increased industry support when supply is vulnerable)
**Store management, governance and decision-making**			
	Management values and leadership	Price gouging, retention of rebates and valuing profits over health	Community concerns and reports that price gouging has been persistent over a long period of time, especially on goods that are in short supply (lack of transparency)
			Store groups retaining supplier rebates rather than rebates going back into the community/store, with higher priced products or stock not required being chosen due to higher rebates
			Stores valuing profits over the health of the community, with prices not appropriate for communities (unaffordability of food), limited promotion of nutrition, enticement of stores to join store groups for profiteering
		Food security and the wellbeing of the community	Store managers not considering the wellbeing of the community, targeting volume of stock in store rather than sales of health stock - “institutional indifference”
			Assumptions made regarding the needs of the community rather than consultation
			Stores valuing nutrition and food security (viewed as a human right, with nutrition policies in place (mostly store management groups), store board desire to implement healthy practices, freight subsidies and cross-subsidised healthy foods (increased accessibility and availability, easing the cost of living)
			Stores/store management groups allocate profits to benevolent activities and hiring of nutritionists, and profits being funnelled back into the community to improve wellbeing

Parent Code			
	**Child Code**	**Themes**	**Subthemes**
		Management training and knowledge of obligations and role	Store group training encompassing the awareness and understanding of a store manager's role in improving health
			Store managers aware of their obligations regarding the health of the community, including land lease agreements and transparency when engaging in projects such as the Market Basket Survey (NT)
			Ambiguity of guidelines resulting in differences in pricing displays, nutrition policies
		Value of Indigenous/community partnerships	Stores valuing solutions led by the community (reliability and honesty) and community feedback, and supporting Indigenous partnerships in supply or procurement (increasing food sovereignty) and Indigenous employment
			Store management groups assisting stores with management rather than taking over, fostering confidence
			Store management groups creating barriers to community being engaged in the day-to-day workings of the store
			No value of Indigenous employment in the store
	Governance/organisational structures	Store governance arrangements impacting merchandising practices	Store management group governance model influencing product range, store layout and pricing
			Choice to join store groups management model increases costs in the short term
			Government owned stores more expensive than privately run stores
			Store group governance model linked to food being less expensive compared to private or community run stores
			Community ownership of stores can create tensions in balancing the viability of store with

Parent Code			
	**Child Code**	**Themes**	**Subthemes**
			affordability
		Having skilled store managers influencing merchandising practice	Correct stock holding by experienced management able to influence product availability in store
	Policy-making and decision-making processes	Store's prioritisation and importance of their connection to community	Involvement of the community and an Indigenous 'voice' to store policies and decisions to empower communities to influence what is sold in the store and how it is sold
			Involvement of communities in store practice has led to successful health-enabling practices in remote stores
		Lack of community involvement with the store and no incentive to have a community influence in decision-making	No involvement of Aboriginal corporations, store committees or community members in decision-making processes, with private stores being profit-driven and no incentive to be involved in the community
			Decisions being made by bookkeepers, store managers and head office instead of community
		Regularity of involvement with key community stakeholders	Regular meetings with store directors (every quarter) guide and give direction to the store, as well as a place to raise community issues
**Store Utilities and Amenities**			
	Economic access to utilities and amenities (store)	High operating costs influencing store practice (prices, availability, access)	High cost of rent from land councils or other private lease (particularly for privately owned stores)
			High cost of refrigeration (upkeep, maintenance, repairs, lost stock if breakdown occurs) especially when reserve stock needed based on travel, accommodation, transport requirements to bring in tradespeople
			Cost of repairs and loss of stock associated with break ins
			High cost of insurance in remote areas prone to cyclones<br>Stores not claiming insurance to avoid increases in premiums

**Parent Code**			
	**Child Code**	**Themes**	**Subthemes**
			Stores not having funds to make large orders to have reserve stockHigh costs of energy/fuel
			High operating costs disproportionately impacting small stores
			Expensive leasing of assets to community stores by store groups
			Costs of meeting regulatory requirements
			Costs of maintaining satellite payment systems
			Despite high operational costs, stores investing in upgrades to increase availability of products
			Costs of insurance/repairs for store manager's accommodation
			Remote stores not receiving the same financial support to offset high operation costs relative to other remote community services or store groups
		Efforts of stores to offset high operational costs	Store efforts to reduce energy consumption
			Efforts of store operators to use resources as efficiently as possible
	Physical access to utilities and amenities (store)	Storage facilities impacting food quality, availability as well as freight costs	Lack of dry and cold storage capacity impacting freight costs, food quality and availability in store, wastage
			Lack of storage for fuel risks running out of electricity at store
			Lack of storage facility impacting the capacity to prepare for extreme weather events or other disruptions (pandemic)
		Consistency of electricity Supply	Power outages in extreme weather events resulting loss of stock, where backup generators not available
		Quality and use of store infrastructure	Undertaking preventative maintenance within stores to prevent major problems

Parent Code			
	**Child Code**	**Themes**	**Subthemes**
			Malfunctioning EFTPOS machines that can take time to replace
			Recent upgrades in fridges allowing for increased promotion of fruits and vegetables
			Poor quality infrastructure in stores (old freezers and fridges)
			Risk in adopting newer tech infrastructure in increased specialisation required for maintenance
			Old infrastructure not energy efficient which drives up power costs
			Repairs and maintenance of refrigerators and freezers is difficult and can take time since not often available locally
		Communications supply (internet use)	Heavy usage of internet by community impacting store access
			Poor internet connection with current satellite system that needs upgrading
			Communications outages causing store to close - community cannot access their money as well as store goods OR store cannot accept transfers from Basics card
		Supply of clean water to store	Inadequate free clean filtered water in communities
**Supply Chain**			
	Cost of freight	High freight costs to remote communities as a driver for decreased accessibility to food/decreased food security	Decreased ability to provide a variety of fresh produce/perishable items in adequate supply due to the cost in cold-chain refrigerated transport, fresh produce often of lower quality on arrival, with short shelf-life
			High freight costs impact the operational costs and viability of the store, impacting frequency of delivery (decreasing availability/accessibility to food)
			High cost of freight increases prices in store (up to +100%)

**Parent Code**			
	**Child Code**	**Themes**	**Subthemes**
			impacting availability and affordability of goods, and increase operational costs for the store
			High freight costs and increased fuel costs being used as an excuse for high prices/price gouging in stores
		Use of freight subsidies/freight contracts	Lack of ability of smaller stores/independent stores to implement a freight subsidy without impacting viability of the store
			Store management groups have the ability to implement freight subsidies on fresh produce, fresh dairy and baby formula to decrease prices.
			Store groups able to implement/share standardised freight costs over the course of the year, and implement contracts with suppliers to drive down freight costs, small stores lack the ability to do this
			Government-owned store groups have cheaper freight costs due to use of government-owned freight companies
			Freight costs impacted by market share
		Freight company operational/additional costs and transport infrastructure impacting costs to remote stores	Increased costs regardless of proximity to regional centres
			Poor road conditions/border restrictions as a result of Covid-19 increase time taken for deliveries, as well as maintenance required for vehicles
			Freight companies on monopolised freight routes increases prices charged to remote stores - only option
			Fluctuating temperatures and use of cold-chain refrigeration increases freight costs
			Freight cost is determined by volume of product, rather than value of the product

Parent Code			
	**Child Code**	**Themes**	**Subthemes**
			Stores that require small volumes to be delivered still pay for a whole vehicle
		Different types of transport used and handling of goods	The cost of sea freight (barge) impacts greatly on cost of delivery, with multiple modes of transport required (truck to get from dock to store). This is sometimes the only option for remote stores.
			High cost of air freight - used to deliver perishables as well as stock during wet season, high costs can decrease availability/access to perishable items during this time
			Use of mutliple forms of transport to deliver to a remote store increases handling of goods, increases opportunities for damage/contamination (including difficulty in maintained safe temperatures) - increases costs associated with food waste, as well as increasing the fees charged for use of ports/lands
			Varied use of transport throughout the year due to seasonal weather events results in varying costs of freight throughout the year
		Resources/suppliers available/abilities of the store to monitor and rectify freight errors	Small/independent stores have decreased ability to monitor for invoicing errors - increased loss
			High cost of freight when returning damaged goods/wrong orders deters stores from doing so
			Use of other stores as suppliers increases freight costs that are payable separate to the goods supplied
	Maintenance and functioning of transport and delivery	Road closures, maintenance, poor conditions and transfer between modes of transport impacting on availability, quality and shelf-life of goods	Poor road conditions causing delays in delivery or extended delivery times, impacting the availability of fresh produce, stores going for long periods of time without stock, shortened shelf-lives on arrival
			Poor road conditions decreasing the quality of goods on arrival due to damages and/or failing

Parent Code			
	**Child Code**	**Themes**	**Subthemes**
			refrigeration
			Long transit/travel times increases spoilage of goods and maintenance required for vehicles
			Handling of goods between different modes of transport can increase chances of contamination/spoilage (barge to road or air to road etc.)
		Functioning of transport and delivery across seasons and weather events	Significant impacts of wet season on road conditions, types of transport used to access communities (increased use of air freight) and damage of goods
			Some communities are unable to be reached during wet season/extreme weather events. Unpredictability of this results in variable supply of goods during this time
			Larger store groups are able to choose suppliers or freight companies that offer alternative forms of transport (e.g. helicopter) at competitive rates, with guarantees of delivery or quality
		Geographical location and smaller market share impacting on options for supply, freight transport and frequency of delivery	Infrequent deliveries (due to small volume of stock and long distance) and the length of time taken to reach remote communities impacts on ability to stock a continuous supply of perishable goods as well as the quality of these goods
			Communities that require charter planes to deliver stock results in less perishable goods being delivered due to lack of cold chain refrigerated transport on the charter
			Panic buying due to Covid-19 and other supply shocks in metropolitan areas can result in increased dfemand for transpot in those areas, limiting options for transport/freight/delivery to remote communities
			Decreased market share of remote communities results in decreased supply of popular products in stores (e.g. iron-

Parent Code			
	**Child Code**	**Themes**	**Subthemes**
			fortified flour)
			Different transport and delivery needs are required for each remote community or store, which results in different challenges
	Industry and supplier relationships and support services	Supplier rebates	Critiques of rebates retained by store group rather than being passed onto customers- while being able to finance high operating costs and financial viability, this process can lack transparency and disadvantage store clients and consumers
			Passing rebates onto consumers able to decrease prices
			Rebates as being able to finance merchandising teams to secure better supply and payment terms
			Reliance on rebates limits suppliers to those that are able to offer larger rebates
		Local suppliers	Remote stores preferencing local suppliers where possible; some larger stores do this even where it is not economical
			Difficulties in sourcing from local suppliers (even where this is preferred): food safety certifications, businesses structures not set up for commercial trading, poor adherence to labelling, inconsistency in quality and quantity
		Unethical supplier conduct	Price gouging
			Raising prices without justification
		Negotiation practices and capacity to ensure secure good quality stock at the lowest price	Benefits of preferred supplier agreements: better prices, better quality food, better quality of service provided
			Sourcing suppliers from outside PSA can increase prices
			PSA protective during COVID-19 disruption

**Parent Code**			
	**Child Code**	**Themes**	**Subthemes**
			Role of merchandising team in negotiating and securing appropriate supply
			Smaller stores not having capacity to seek alternative suppliers and are are more vulnerable to price increases
			Supply chain disadvantages (location, price, volume, negotiating capacity) relative to supermarkets
			Larger wholesalers offering better service than smaller suppliers
			Benchmarking utilised as a method to compared between supplier arrangements
			Concern that store management groups consolidated supply chain precludes securing best price option
		Limited access to supply of certain foods	Limited access to certain foods (including generic products) from suppliers
			Supplier price increases during COVID meaning product weren't stocked
		Support from suppliers/ manufacturers/ wholesalers	Suppliers/ manufacturers providing discounts and promotions
			CSR donations and partnerships towards wellbeing initiatives
			Lack of marketing/ resources provided by suppliers relative to urban/ large supermarkets
			Suppliers providing refund if product arrives unfit for sale
		Utilising supermarkets as suppliers (+ve/ -ve)	Small stores having to source from supermarkets can contribute to meeting demand from store
			Small stores sourcing from supermarkets increases prices further and can mean there is limited access to certain products (purchase limits), little support to ordering stock or efficiency for these stores offered by supermarkets

Parent Code			
	**Child Code**	**Themes**	**Subthemes**
	Supply chain planning and risk mitigation	Capacity of stores to build up stock reserves ahead of wet season	Store building up stock of non perishable items to mitigate against wet season disruption
			Lack of cold storage infrastructure impacting capacity of stores to take on additional stock
			Stores committing to improving storage infrastructure to mitigate against future disruption
			Stores lacking financial stability to secure stock reserves in advance of wet season
			Store groups financially underpinning smaller stores to ensure freight costs and stock reserves can be covered without passing on prices to customers
			Not planning for annual wet season disruption leading to stores running out of food and community going without food. One reference linked this to community unrest
		Shifting to air freight	Stores shifting to air freight during wet season which can increase prices for stores that cannot absorb cost
			Stores reducing quantity of perishable foods ordered during wet season to reduce air freight costs
		Coordination between supply chain actors to secure stock	Consolidated purchasing arrangements to reduce freight costs
			Collaboration between supply chain actors (including governments in some instances) to ensure remote stores are adequately stocked
			Coordinating between stores (within same store group) to shift stock where it's needed most
			Inefficiency with remote store supply chain networks (procurement, warehousing, transport and distribution, communications, information systems, ordering) as a contributor to remote food insecurity

Parent Code			
	**Child Code**	**Themes**	**Subthemes**
		Challenge of coordinating ordering ahead of disruptions	Independent stores struggled to mitigate impacts of COVID-19 on supply of essential goods
			Despite the store's best efforts in ordering ahead of time, being cut off from supply chains, difficulty in coordinating freight and a delay between ordering and receiving stock can mean that stores do run out of items. Occasionally alternative supply routes can also be affected (Townsville flood example).
		Adapting ordering/ pricing practices (other than ordering additional stock)	Some stores reducing delivery frequency to reduce costs
			Trend in stores increasing delivery frequency over time
			Sourcing alternative/ substitute items in face of supply chain disruption and anticipating changes in consumer purchasing behaviour when ordering
			Avoid stocking items that have increased in price due to weather events/ other disruption
			Flexibility with suppliers/ wholesalers able to contribute to securing adequate supply
			Budgeting for disruptions and smoothing prices out across the year to avoid altering prices after natural events
	Store context influencing supply chain dynamics	Size and location/proximity (to other communities) of community impacting viability, variety of foods	small community size impacting the viability of a store, with stores acting as essential services (only store within the region) rather than profitable businesses, driving prices up (-ve)
			Smaller community size limits the amount and variety of foods available in the store
			Smaller community size negatively affects access to physical amenities for stores, including size of storage facilities
			Store groups have the ability to support smaller, unviable stores to deliver essential services (in small communities)

Parent Code			
	**Child Code**	**Themes**	**Subthemes**
			Small stores in small communities using major retailers (Woolworths, Coles) as suppliers (buying off-shelf) can impact the volatility of supply. Major retailers are not set up to supply remote communities and this can result in inadequate supply
			The remote location of stores resulting in lack of access to suppliers and goods, as well as an insecure, stressed supply chain (this is the case even when there is a major supermarket nearby e.g. Nhulunbuy)
		Competition and market power	Lack of competition driving poorer availability of goods to communities, lack of innovation in store practices and less promotions (higher, unregulated prices)
			Decreased competition as a result of remote location (higher freight costs, impacts viability of stores)
			Market power of major retailers (Coles, Woolworths) impacting on supplier relationships and availability to remote stores, with small stores having difficulty obtaining stock and experiencing a volatile supply chain.
			Market power influencing funding available, with small, independent stores having less access to funding or supplier rebates
			Competition in remote communities (especially privately owned) has an impact on the ability of the other store/s to implement in-store nutrition interventions as they have a greater ability to decrease the viability of the store (community members have another store to purchase their goods from)
			Lack of competition creates inelasticity of demand from community, meaning a store can provide inferior products/services without consequence
		Purchasing power of stores	remote stores have poor purchasing power resulting in

Parent Code			
	**Child Code**	**Themes**	**Subthemes**
			uneconomical (not being able to bulk-buy)/disadvantaged (not being able to access promotions from suppliers) relationships with suppliers, with small stores taking more time (a valuable resource) to build beneficial relationships with suppliers (-ve) and impacts what suppliers are being utilised/chosen
			Store groups/small stores within the same area creating a conglomerate have increased purchasing power and more affordable pricing (+ve)
			Poor purchasing power of remote stores negatively impacts on their ability to implement any pricing interventions to improve nutrition
			poor purchasing power resulting in a decreased variety of goods (including the core variety of healthy foods) available in store, as well as the ability to pre-purchase stock to guarantee a continuous supply and impacts what is being stocked (e.g., not stocking fresh meat due to the inability to store a viable amount)
		Economies of scale and operating costs	larger stores/store groups and stores that have created conglomerates are able to lower operating costs (rent, freight costs (+ coordinating larger deliveries)) in order to decrease food prices (even in smaller, unviable stores that have gained support from a store group) and can have more opportunity for relationships with suppliers
			Small remote stores do not have the ability or resources to produce/create own brands or take advantage of generic products
			Smaller economies of scale can result in a decreased frequency of delivery, which in turn decreases the availability of food to the community, and decreased quality of the food that is available.
		Remoteness of stores impacting on application of	Consumer law or regulation is not applied in remote settings, limited

Parent Code			
	**Child Code**	**Themes**	**Subthemes**
		regulation	control regarding the pricing of goods
**Workforce**			
	Workforce investment, training and capacity building/skill development	Capacity for nutrition training of staff	Lack of nutrition-related training due to lack of skilled staff to be able to deliver the training, which in turn creates a difficult environment to sustainably implement any nutrition interventions
			Store groups have the capacity to employ in-house public health nutritionists that have the skills to train store staff in nutrition
			the public health nutrition workforce lacks positions for Indigenous nutritionist in the remote store space
		Opportunities/barriers for/to local Indigenous employment	store groups providing more opportunities for local Indigenous employment (compared to privately owned stores), as well as training opportunities such as certification and apprenticeships
			store group has well-established pathways for local employment, with management being trained as role models
			Language barriers, poor numeracy and literacy skills and lack of cultural training add another dimension to hiring and training local staff. Hands-on, face-to-face training is preferred, which can take more time that conventional training such as written courses
		Skill-set of store management to respond to diverse operations of remote community stores	Managers are hired based on their ability to run a business rather than their ability to foster good nutrition
			Lack of cultural training for store management results in high management turnover
			lack of training regarding advocacy or applying for funding resulting in lack of funding for stores

**Parent Code**			
	**Child Code**	**Themes**	**Subthemes**
			management not being sufficiently trained at the beginning of their placement (have come from metropolitan retailers) and therefore do not run the remote store sufficiently
			Committee training to ensure staff are all adequately trained in pricing and supply and can adequately train other staff
		Training capacity, costs and opportunities	Limited training or capacity building opportunities within a store group
			Training in remote communities comes with a high cost due to external trainers travelling into communities
		Access to training resources	limited access to training
			store groups adopting the same training and management courses as major retailers ensures consistency
			Researchers aiding in set-up and training of staff to implement nutrition interventions in-store can add to their sustainability (increased resources for training)
	Workforce stability and staffing	Staff turnover impacting relationships, sustainability and operational costs	High management turnover creating unsustainable interventions
			High management turnover creating fractured relationships with community and other store staff
			Good staff stability within a store group (n=1)
			Store groups recruiting staff from other store groups, increasing staff turnover
			Higher costs to ensure staff retainment (higher wages, housing costs covered)
		Staffing and impact on store operations (trading hours, staff skills, staff retainment)	Inadequate staffing resulting in decreased trading hours
			Adequate staffing in large store groups allowing for stable merchandising practices

Parent Code			
	**Child Code**	**Themes**	**Subthemes**
			Limited availability of skilled/relevant staff to be employed by the store
			Inadequate staffing resulting in lack of capacity to participate in interventions
			Inadequate staffing as a result of cultural/familial obligations (e.g., childcare, family unrest, humbugging preventing employment at the store)
			Western services or store operations not conducive to retainment of Indigenous employment
			Management using store operations/store trading hours as leverage for control in the community (store does not open if Indigenous workers do not turn up)
		Nutrition staff/public health nutrition workforce capacity (remote stores)	Lack of public health nutrition workforce employed to work with remote stores
	Value and support felt by staff in the workplace and community	Lack of respect for store staff	lack of respect from community (staff assault)
			lack of respect from higher management (concerns/suggestions not being listened to)
		Cultural and familial obligations hindering employment within the store	no support from management with regard to other obligations (e.g., childcare, funerals)
			community/family pressuring staff to make decisions that may jeopardise their employment (e.g., humbugging)
	Staff/retailer support of store	Integral relationships with the community	Stores being a driver of change within the community and thus, being an integral part of the community and its structure
	Cost of staffing	Additional costs of remote staffing/management	Higher wages needed to attract qualified staff to remote communities, creating higher prices or the ability to allocate funding to other areas/services of the store (e.g. charitable activities)

Parent Code			
	**Child Code**	**Themes**	**Subthemes**
			Higher costs to retain staff (providing housing, vehicle, utilities, increased leave entitlements and relief staff, travel to and from communities, includes impact of Covid-19 and quarantine/travel requirements). This can impact the profitability of the store, and impact ability to implement health interventions
		Differences in the burden of the cost of staffing for different store governance structures	The use of supplier rebates/access to supplier rebates helps to fund merchandising staff in store groups
			cost of staffing being the major contributor to operating costs, especially in independent stores, which in turn creates higher prices
		Cost of training staff	Training staff to suit remote community stores has high costs associated, and low retainment of staff can result in losses for the store (skilled workers move onto other stores)
			Training staff with language barriers, low numeracy or literacy skills increases costs even further, and subsidies provided to do so do not cover costs

Supplementary Table 3: Worked question matrix

**PROPOSED QUESTION**	**Guideline 1: Can store managers directly speak to this issue?**	**Guideline 2: Does this theme arise often in the data?**	**Guideline 3: Will this question help to capture the range of experiences in remote stores?**	**Guideline 4: Is this question specific enough to be able to act on/understand the issue?**	**Guideline 5: Is this information that can come from another part of the project?**
To what extent do you agree that your store management has the skills, power or network of stakeholders to be able to advocate for healthy store practice?	Y	N (<5 mentions)	Y	Y	N

PROPOSED QUESTION	Guideline 1: Can store managers directly speak to this issue?	Guideline 2: Does this theme arise often in the data?	Guideline 3: Will this question help to capture the range of experiences in remote stores?	Guideline 4: Is this question specific enough to be able to act on/understand the issue?	Guideline 5: Is this information that can come from another part of the project?
To what extent do you agree that lack of healthy store practice in other stores (in or outside of your community) make it hard for your store to implement healthy store practice?	Y	N (<5 mentions)	Y	Y	N
To what extent do you agree that nutrition messaging of services in the community align with your store's healthy store practice?	Maybe	Y (>10 mentions)	Y	Y	N
To what extent do you agree your store has the capacity to fully implement food safety policies to support healthy store practice?	Y	N (<10 mentions)	Y	Y	N
To what extent do you agree the following factors are a barrier to food safety and thus, healthy store practice?<br>a) adequate labour/staffing<br>b) training and knowledge/skills of staff<br>c) adequate infrastructure of the store (e.g., storage facilities, sinks, refrigeration)	Y	N (<5 mentions)	Y	Y	N
To what extent do you agree that your store has the knowledge/skills to access, collect and use data (own sales data, price data from other stores etc.) to make informed decisions about healthy store practice?	Y	Y (>10 mentions)	Y	Y	N
To what extent do you agree that your store is effectively sharing information with the community about your healthy store practice?	Y	Y (>10 mentions)	Y	N (may need more information around WHY if there isn't effective sharing of information?)	N
To what extent do you agree that your store has a positive relationship with the community that enables healthy store practice?	Y	Y (>10 mentions)	Y	Maybe (doesn't necessarily give a clear response to take action to improve a relationship)	N

PROPOSED QUESTION	Guideline 1: Can store managers directly speak to this issue?	Guideline 2: Does this theme arise often in the data?	Guideline 3: Will this question help to capture the range of experiences in remote stores?	Guideline 4: Is this question specific enough to be able to act on/understand the issue?	Guideline 5: Is this information that can come from another part of the project?
To what extent do you agree that the demand from the community influences your healthy store practice, including the availability/variety and promotion of the following foods?<br>a) healthy foods<br>b) unhealthy foods	Y	Y (>30 mentions)	Y	Y	N (?could compare response against healthiness score)
To what extent do you agree that the time provided to your store by public health nutritionists is adequate to help your store's healthy store practice?	Y	Y (>10 mentions)	Y	Y	N
To what extent do you agree that working with other services in the community helps your store to implement and maintain healthy store practice?	Y	N (<5 mentions)	Y	Y	N
To what extent do you agree that the following partners help your store to implement and sustain healthy store practice?<br>a) community services (clinic, school)<br>b) government (federal, state/territory, local)<br>c) industry/suppliers (e.g., Coca-Cola Amatil, Metcash)<br>d) regulators (e.g., stores licensing regulator, ACCC)	Y	Y (>10 mentions)	Y	Y	N
To what extent do you agree that your store always considers food affordability alongside profit when making decisions regarding healthy store practice?	Y (if pricing decisions are made by the store manager)<br>N (if pricing decisions are made by a store management group/store board)	Y (>30 mentions)	Y	Y	Y (?policy)
To what extent do you agree that the governance structure of your store helps your store implement healthy store practice?	Y	N (<10 mentions)	Y	Y (if compared against demographic data on the governance structure of the store)	Y

PROPOSED QUESTION	Guideline 1: Can store managers directly speak to this issue?	Guideline 2: Does this theme arise often in the data?	Guideline 3: Will this question help to capture the range of experiences in remote stores?	Guideline 4: Is this question specific enough to be able to act on/understand the issue?	Guideline 5: Is this information that can come from another part of the project?
To what extent do you agree that the following groups of people are given a 'voice' to guide your store's healthy store practice?<br>a) community members<br>b) board of directors, store committee	Y	Y (>10 mentions)	Y	Y	N
To what extent do you agree that the following operational costs make it hard for your store to implement healthy store practice?<br>a) cost of maintenance and repairs for store infrastructure (e.g., refrigeration)<br>b) cost of rent/ lease agreement<br>c) costs of repairs from break-ins (e.g., fixing a broken window)<br>d) costs of electricity and fuel to run the store	Y	Y (>30 mentions)	Y	Y	N
To what extent do you agree that lack of storage facilities make it hard for your store to implement healthy store practice?	Y	Y (>10 mentions)	Y	N (maybe need to be more specific to capture the influence of the storage facilties?)	N
To what extent do you agree that disruptions in the following make it hard to implement healthy store practice?<br>a) electricity<br>b) internet<br>c) water	Y	Y (>10 mentions)	Y	Y	N
To what extent do you agree that your store has adequate (and well-functioning) infrastructure (e.g., fridges, freezers) to implement healthy store practice?	Y	Y (>10 mentions)	Y	Y	N
To what extent do you agree that high freight costs make it hard for your store to implement healthy store practice?	Y	Y (>30 mentions)	Y	Y	N
To what extent do you agree that the cost to send goods back to a	Y	N (<10 mentions)	Y	Y	N

PROPOSED QUESTION	Guideline 1: Can store managers directly speak to this issue?	Guideline 2: Does this theme arise often in the data?	Guideline 3: Will this question help to capture the range of experiences in remote stores?	Guideline 4: Is this question specific enough to be able to act on/understand the issue?	Guideline 5: Is this information that can come from another part of the project?
supplier makes it hard for your store to implement healthy store practice?					
To what extent do you agree that road closures or poor road conditions make it hard for your store to implement healthy store practice?	Y	Y (>30 mentions)	Y	Y	N
To what extent do you agree that the frequency of delivery of goods makes its hard for your store to implement healthy store practice?	Y	Y (>30 mentions)	Y	Y	N
To what extent do you agree that your store's capacity to negotiate with suppliers helps your store to implement healthy store practice?	Y	Y (>20 mentions)	Y	N (may not capture if a store HAS capacity, only whether it is helpful?)	N
To what extent do you agree that your store's relationship with, and support from manufacturers and suppliers helps your store to implement healthy store practice?	Y	Y (>10 mentions)	Y	Y	N
To what extent do you agree that your store has the capacity to plan for and respond to expected (wet season) and unexpected events (e.g., natural disasters, COVID-19) that can impact healthy store practice?	Y	Y (>30 mentions)	Y	N (broadly indicate if stores need more assistance in resilience planning or disaster preparedness but no more specific than that)	N
To what extent do you agree that your store has the capacity to come together with other stores or communities to increase buying power or market power for your store to implement healthy store practice?	Y	Y (>30 mentions)	Y	Y	N (?demographics data)
To what extent do you agree that business competition impacts on your store's healthy store practice?	Y	Y (>10 mentions)	Y	Y	N
To what extent do you agree your store	Y	N (<10 mentions)	Y	Y	N

PROPOSED QUESTION	Guideline 1: Can store managers directly speak to this issue?	Guideline 2: Does this theme arise often in the data?	Guideline 3: Will this question help to capture the range of experiences in remote stores?	Guideline 4: Is this question specific enough to be able to act on/understand the issue?	Guideline 5: Is this information that can come from another part of the project?
management is equipped with the resources (e.g., finances, time) you need to train staff on healthy store practice?					
To what extent do you agree that the longer a store manager has been in the community, the easier it is for your store to implement healthy store practice?	Maybe	Y (>10 mentions)	Y	Y	N
To what extent do you agree that your store has adequate workforce/staffing capacity to implement healthy store practice?	Y	Y (>10 mentions)	Y	Y	N
To what extent do you agree that your staff are valued or seen as a driver of change for better nutrition within the community?	Maybe	N (<5 mentions)	Y	Y	N
To what extent do you agree that staffing expenses at your store (e.g., wages, training and provision of housing for staff) make it hard for your store to implement healthy store practice?	Y	Y (>10 mentions)	Y	Y	N
To what extent do you agree that community members' lack of access to electricity for refrigeration, water and storage space for food in their homes makes it hard for your store to implement healthy store practice?	Maybe	Y (>30 mentions)	Y	Y	N
Y; Yes, N; No.					

Supplementary Table 4: Survey Tool

Healthy in-store operations includes operations related to:

- Product: availability, shelf-space, range, quality;

- Placement: location, end-of-aisles, front of the store, near the checkout;
- Price: specials, subsidies;
- Promotion: shelf-talkers, posters, stickers, branding.

We are interested in how these factors may influence your store's healthy in-store operations, at this point in time.

Question Number	Preamble	Question (How much do you agree with…)
**1**	*Different healthy in-store operations in other stores or communities can affect what you do in your store because of worry about losing customers and impact on the business.*	It is hard for my store to put in place healthy in-store operations if other stores do not do the same.
**2**	*The size of a store and how close they are to other stores can possibly improve a store's buying power due to the amount of stock ordered. Building good relationships and negotiating with suppliers can result in better prices and service.*	Your store is able to come together with other stores or communities to increase buying power, helping your store to carry out healthy in-store operations.
**3**	*Nutrition messages and activities in the community (in places like the school, clinic, police station) can affect a store's healthy in-store operations.*	The nutrition messages of different organisations in the community are the same as those your store conveys to customers through your healthy in-store operations.
**4**	*When groups of stakeholders work together to support changes to the community's nutrition, it can affect a store's healthy in-store operations.*	The following groups make it easier for your store to carry out healthy in-store operations.<br>• Community services (e.g., clinic, school)<br>• Regulators (e.g., stores licensing regulator, ACCC)<br>• Government (federal, state/territory, local)<br>• Industry/suppliers (e.g., Coca-Cola Amatil, Metcash)
**5**	*Relationships between the store and community members, as well as what is happening in the community can affect healthy in-store operations.*	Your store management has a good relationship with the community that makes it easier to carry out healthy in-store operations.
**6**	*Data (such as showing how well products are selling or showing the cost of food) can guide healthy in-store operations.*	Your store has the knowledge/skills to access, collect and use data to make decisions about healthy in-store operations.
**7**	*Sharing information about the store, in a way that is easy to understand and celebrates positive changes, can help to build a strong relationship with the community.*	Your store has good communication with the community about healthy in-store operations.
**8**	*There are a range of factors in a remote community that affect a store's ability to carry out healthy in-store operations including the people that are given a 'voice' to set and guide policy.*	These groups of people are given a 'voice' to guide your store's healthy in-store operations.<br>• Community members<br>• Board of Directors, Store Committee
**9**	*Food security means always having access to enough nutritious and culturally appropriate food at an affordable price. Healthy in-store operations such as fruit and vegetable subsidies, ongoing price discounts on healthy food and drinks help with food affordability.*	Your store thinks about food affordability when making decisions about the business and profit.

**10**	*Remote stores can have barriers such as long freight journeys, unreliable electricity and high staff turnover resulting in high running costs, which can affect healthy in-store operations*.	The following operational costs are barriers for your store to carry out healthy in-store operations.<br>• Cost of freight<br>• Cost of maintenance and repairs (e.g., fridges)<br>• Cost from break-ins (including store closure and repairs)<br>• Cost of rent/lease agreement<br>• Costs of electricity and fuel to run the store<br>• Cost of staffing (e.g., wages, training and housing for staff)<br>•
**11**	*Working utilities (such as electricity), facilities and appliances (such as fridges, storerooms) help a store to carry out healthy in-store operations. Please think about this point in time.*	Lack of storage space to store extra stock of food and drinks affects your store's ability to carry out healthy in-store operations.
**12**	as above	Lack of working fridges and freezers, among other appliances, affects your store's ability to carry out healthy in-store operations.
**13**	as above	Disruptions in electricity (power outages, unreliable electricity) impacts your store's healthy in-store operations.
**14**	as above	Disruptions in the internet (slow internet, internet outage) impacts your store's healthy in-store operations.
**15**	*The maintenance of roads and vehicles, and the quality of the food and drink when they are delivered, can impact healthy in-store operations.*	Road closures or poor road conditions are barriers for your store to carry out healthy in-store operations.
**16**	*Remote stores often have deliveries less often than urban stores, due to remote location and high freight costs. This can affect the foods and drinks that are stocked, and therefore healthy in-store operations.*	The frequency of delivery of fresh produce (fruits, vegetables, dairy) is a barrier for your store to carry out healthy in-store operations.
**17**	*During unexpected events (such as COVID-19 or cyclones) and expected events (such as the wet season), getting to remote communities can be difficult. It can change what types of food and drinks are available in the store and therefore healthy in-store operations.*	Your store is able to adequately plan for and respond to events that impact healthy in-store operations.
**18**	*The availability of staff, changes in staff and varying staff skills in nutrition can impact a store's healthy in-store operations.*	Your store has enough staff with the right skills to carry out healthy in-store operations.
**19**	*Customer demand for food and drinks can influence what food and drinks are stocked in a store, or what products are promoted.*	The demand from the community for unhealthy foods is a barrier to your store's healthy in-store operations.
**20**	*Public health nutritionists (from health organisations, government or internal nutritionists) can support remote stores to carry out healthy in-store operations.*	The time provided to your store by public health nutritionists is adequate to support your healthy in-store operations.
**21**	*Not applicable*	How often does your store have electricity disruptions or outages?
**22**	*Not applicable*	How often does your store have internet disruptions or outages?

**23**	*Not applicable*	How often does your store experience break-ins?
**24**	*Not applicable*	On average, how often is your store impacted by road closures or poor road conditions?
**25**	*Not applicable*	How often does your store get deliveries of fresh produce (fruit, vegetables and dairy)?
**26**	*Not applicable*	How often does your store receive support from a public health nutritionist (from a health organisation, government or your store group)?
**End**	*Not applicable*	Are there any other factors that have a big impact on your store's ability to carry out healthy in-store operations that have not been covered? Please write them below:

Scale for question responses; Strongly agree, agree, neither agree nor disagree, disagree, strongly disagree. Questions 21-26 refer to frequency of occurrence.
